# oPOSSUM-3: Advanced Analysis of Regulatory Motif Over-Representation Across Genes or ChIP-Seq Datasets

**DOI:** 10.1534/g3.112.003202

**Published:** 2012-09-01

**Authors:** Andrew T. Kwon, David J. Arenillas, Rebecca Worsley Hunt, Wyeth W. Wasserman

**Affiliations:** * Centre for Molecular Medicine and Therapeutics, Child and Family Research Institute, University of British Columbia, Vancouver, British Columbia, V5Z 4H4, Canada; † Department of Medical Genetics, University of British Columbia, Vancouver, British Columbia, V6T 1Z4, Canada; ‡ Genetics Graduate Program, University of British Columbia, Vancouver, British Columbia, V6T 1Z4, Canada; § Bioinformatics Graduate Program, University of British Columbia, Vancouver, British Columbia, V6T 1Z4, Canada

**Keywords:** gene regulation, ChIP-Seq, transcription factor binding sites (TFBS), transcription

## Abstract

oPOSSUM-3 is a web-accessible software system for identification of over-represented transcription factor binding sites (TFBS) and TFBS families in either DNA sequences of co-expressed genes or sequences generated from high-throughput methods, such as ChIP-Seq. Validation of the system with known sets of co-regulated genes and published ChIP-Seq data demonstrates the capacity for oPOSSUM-3 to identify mediating transcription factors (TF) for co-regulated genes or co-recovered sequences. oPOSSUM-3 is available at http://opossum.cisreg.ca.

The properties of cells within an organism are defined by a complex interplay between proteins, RNA, and the genome, which can be conceptualized as the gene regulatory network. Two important components of the gene regulatory network are the DNA-binding *trans*-acting transcription factors (TF) and their corresponding transcription factor binding sites (TFBS) in the DNA. Sets of proximal TFBSs that are sufficient to cooperatively mediate TF-regulated patterns of expression constitute *cis*-regulatory modules (CRM). CRMs are the scaffold for combinatorial TF interactions, enabling a limited number of sequence-specific DNA binding TFs to participate in an exponential number of combinations, each potentially conferring specific patterns of gene activity (Arnone and Davidson 1997).

In studying gene regulation within a cell or tissue, researchers are commonly confronted with the need to analyze sets of genes sharing a characteristic, such as co-expression, as they seek to infer properties of the gene regulatory network. A significant insight into the regulatory network structure is obtained when the mediating TFs for the observed expression patterns are identified. A key strategy in genome biology for determining such TFs is to determine the sequence motifs that are over-represented in the *cis*-regulatory regions relative to some control. The successful predecessors to oPOSSUM-3, oPOSSUM (Ho Sui *et al.* 2005) and oPOSSUM-2 (Ho Sui *et al.* 2007), were developed to identify statistically over-represented, predicted TFBS in co-regulated gene sets. Two complementary scoring methods measured the over-representation: (1) Z-scores based on normal approximation to the binomial distribution that measures the change in the relative number of TFBS motifs in the foreground gene set compared with the background set, and (2) Fisher scores based on a one-tailed Fisher exact probability assessing the number of genes with the TFBS motifs in the foreground set *vs.* the background set. Using the JASPAR database as the source of DNA binding profiles (Portales-Casamar *et al.* 2010), the original oPOSSUM was designed to identify over-represented TFBSs, later referred to as Single Site Analysis (SSA). The original system also incorporated a conservation filter using phylogenetic footprinting based on pairwise alignments of orthologous sequences from human and mouse. In oPOSSUM-2, an additional analysis method called Combination Site Analysis (CSA) was introduced to identify over-represented proximal pairs of TFBSs. Separate oPOSSUM-2 implementations were released for two additional model organisms (*C. elegans* and *S. cerevisiae*). The nematode oPOSSUM-2 database was based on alignments between *C. elegans* and *C. briggsae*. The oPOSSUM-2 yeast system did not incorporate conservation filters, as the compact nature of the yeast genome results in dramatically reduced search space and noise compared with larger genomes. The oPOSSUM software is a highly cited tool for TFBS motif over-representation analysis (as assessed by Google Scholar citation counts), perhaps due to the ease of use and power of the approach. On average, excluding automated internet search software, 340 unique users work with oPOSSUM-2 each month.

Since the release of the original oPOSSUM system, a plethora of TFBS over-representation analysis tools have been introduced. TOUCAN2, a workbench system for regulatory sequence analysis implemented by Aerts *et al.* (2005), contains features for identifying over-represented TFBS in proximal promoters of co-regulated genes. Defrance and Touzet (2006) developed the TFM-Explorer, which assesses conservation of spatial arrangements of regulatory elements. Promoter Analysis Pipeline by Chang *et al.* (2007) includes TFBS identification in gene sets as a component of the workbench, using non-redundant profiles from public databases. Piechota *et al.* (2010) developed the cREMaG database, which attempts to correct for the confounding influence of variable information content of TFBS profiles, distinguishes between constitutive and inducible transcriptional forms of genes and reports the presence of CpG islands. Many of the methods provide web-based user interfaces, some of which are maintained. oPOSSUM-2 was found to perform well in an independent assessment of motif over-representation analysis tools (Meng *et al.* 2010).

Since the implementation of these approaches, technology changes have greatly affected regulatory sequence studies. First, comprehensive multi-species sequence comparison measures are conveniently available in the form of phastCons and phyloP scores from the UCSC genome databases (Hubisz *et al.* 2011). Phylogenetic footprinting, used by many TFBS enrichment programs, when performed with pairwise sequence alignments places emphasis on the quality of the choice of organism with which to compare and can sharply limit the number of genes that can be analyzed. Compared with pairwise alignments, multi-species analysis improves the quality of sequence alignments (Kumar and Filipski 2007) and greatly increases the number of genes available for analysis. The proliferation of large-scale regulatory sequence profiling methods such as ChIP-Seq has demonstrated that TF-DNA interactions frequently occur outside of conserved regions (Schmidt *et al.* 2010). Genomic regions bound by TFs in ChIP experiments are a snapshot of a single cell-type and set of conditions, and not all regions are necessarily functional *cis*-regulatory sequences. In the absence of or in complement with experimental data, conservation is a useful filter for enabling computational motif enrichment analysis. Second, there has been a major update to the JASPAR database, an open-source, non-redundant, curated repository of TFBS profiles (Portales-Casamar *et al.* 2010). The update provides a significant increase in non-vertebrate profiles, permitting the extension of regulatory analysis software to many non-vertebrate species, such as insects. Third, widespread application of ChIP-Seq profiling has resulted in an explosion of the number of potential regulatory sequences to be analyzed (Johnson *et al.* 2007; Malhotra *et al.* 2010; Schmidt *et al.* 2010). These experiments produce sets of TF bound and control sequences, in which the foreground target (TF-bound) sets purportedly contain regulatory signatures of interest, whereas the background (control) sets lack those features. Such data are optimal for TFBS over-representation analysis, creating strong demand for a new generation of software that allows analysis from both a sequence-based perspective and a gene-based perspective.

Here we describe oPOSSUM-3, a system that capitalizes upon the aforementioned research developments. The new system features a panel of upgraded and novel approaches to regulatory sequence analysis, including Single-Site Analysis (SSA) and anchored Combination-Site Analysis (aCSA) (Figure 1). A novel extension of the system addresses the challenge imposed by homologous TFs with highly similar (or identical) binding specificity. Such profile similarity will be of increasing impact on motif enrichment analysis as the number of TF profiles depicting similar binding grows. The TFBS Cluster Analysis (TCA) and anchored Combination TFBS Cluster Analysis (aCTCA) present results focused on TFBS sequence patterns rather than individual profile names. This new approach to regulatory sequence motif over-representation analysis has been assessed against reference sets of co-regulated genes and large-scale ChIP-Seq sequence collections. Assessments against reference cases exemplify the utility of oPOSSUM-3 for the identification of mediating TFs. The new system should maintain the oPOSSUM service as a popular resource for motif over-representation analysis.

**Figure 1:**
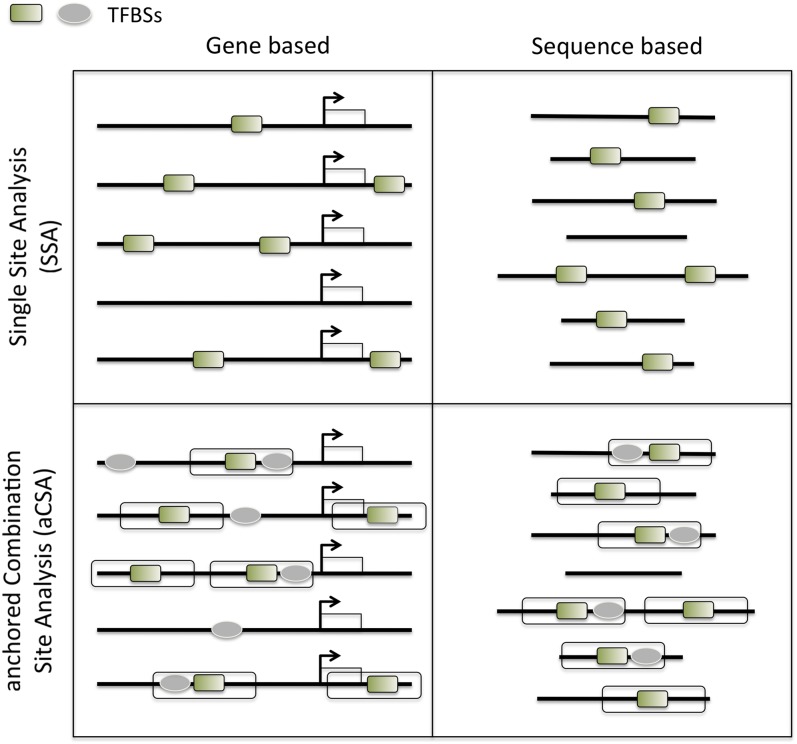
Overview of the main analysis types available in oPOSSUM-3. The input for oPOSSUM can be either gene-based, which makes use of pre-computed results based on annotated genomic information, or sequenced-based, in which the user supplies the input sequences (*e.g.* ChIP-Seq results) for analysis. There are four methods available: (1) Single Site Analysis (SSA), (2) TFBS Cluster Analysis (TCA), (3) anchored Combination Site Analysis (aCSA), and (4) anchored TFBS Cluster Analysis (aCTCA). The first two methods apply enrichment analyses to individual TFs or TFBS clusters, whereas the latter two methods apply enrichment analyses to pairs of individual TFs or pairs of TFBS clusters. SSA and aCSA are depicted in this figure.

## Materials and Methods

### Nomenclature

Throughout this article, we refer to TFs and genes using a capitalized first letter followed by lowercase letters; all suffix characters are capitalized (*e.g.*, Mef2A).

### Data sources

Gene and transcript annotation and genomic sequences were retrieved from Ensembl v64 except for *C. elegans*, which uses v54 for compatibility with conservation scores provided by the UCSC Genome Browser (Flicek *et al.* 2010). For *C. elegans*, operon annotations were retrieved from Wormbase WS200 (Harris *et al.* 2010). The phastCons scores were retrieved from the UCSC Genome Browser, based on the following score sets: (1) for human (hg19), phastCons46wayPlacental; (2) for mouse (mm9), phastCons30wayPlacental; (3) for fruit fly (dm3), phastCons15way; and (4) for nematode (ce6), phastCons6way. As UCSC ce6 database for *C. elegans* is based on WS190, genomic regions that had insertions or deletions between WS190 and WS200 were excluded. All “known” genes in the Ensembl databases were included in the database. For TFBS profiles, the 2010 release of the JASPAR database was used; all profiles from the CORE and PBM collections were included. A custom profile collection (referred to as PENDING), which is not included in JASPAR, was implemented to include profiles of interest for our analysis (Figure S1).

### Calculation of TFBS motif over-representation

Two statistical measures are used to determine the TFBS motifs that are over-represented in the foreground set *vs.* the background set, representing two different models for the occurrences of TFBSs. For sequence-based analysis, a third statistical measure of motif centrality is calculated.

#### Z-scores:

The Z-score is used to assess whether the rate of occurrence of a given TFBS in the foreground sequence set differs significantly from the expected rate calculated from the background set based on a simple binomial distribution model. For a given TFBS, let *X* denote the number of predicted binding site nucleotides in the foreground sequence set, and *B* the number of predicted binding site nucleotides in the background set. If *n* is the total number of nucleotides in the foreground set, and *N* the total number for the background set, the expected value of *X* is given by *µ = BC*, where *C = n/N*. The probability of success is given by *P = B/N*, and the standard deviation is $\sigma =\sqrt{nP(1-P)}$. Then, if *x* is the observed number of binding site nucleotides in the foreground set, using the normal approximation to the binomial distribution with a continuity correction of 0.5, the Z-score can be calculated as *Z = (x−µ−0.5)/σ*. The continuity correction is applied to correct for the normal distribution being continuous while the binomial distribution is discrete. The continuity correction (0.5) is subtracted from *x* because we are interested in the probability that *x* is strictly less than a given value. The calculation was implemented in the R statistics package, as were the subsequent enrichment scores (R Development Core Team 2008).

#### Fisher scores:

The Fisher probability test is used to determine the probability of a non-random association between the foreground sequence set and a given TFBS by comparing the proportion of foreground sequences containing a given TFBS with the proportion of background sequences with that site. The Fisher probability is calculated using a hypergeometric probability distribution, which describes sampling without replacement from a finite population consisting of two types of elements. The Fisher scores are obtained by taking the negative logarithm of the probabilities (natural logarithm is used). In contrast to the Z-score, only the presence or absence of a TFBS in a given sequence is considered; the number of occurrences of a TFBS is not included in the probability calculation.

#### Centrality KS scores:

For sequence-based analysis methods, the Kolmogorov-Smirnoff (KS) test is used to compare the empirical distributions of the TFBS locations between target and background sets. In ChIP-Seq experiments, the highest number of sequence tags under a peak region is expected to occur where there is specific binding by the target TF to the DNA, such that functional TFBSs are located at or in close proximity to this maximum confidence position (MCP). Thus, in target sequences, the distances of the target TFBSs to the MCP (which we refer to as the DistMCP) are expected to be clustered around zero, whereas in background sequences, the binding sites would be distributed randomly. An example is given in Figure S2, which shows the binding site distributions in the Nfe2L2 ChIP-Seq dataset by Malhotra *et al.* (2010). Van Helden *et al.* (2000) employed such position analysis for detecting 3′ signals in yeast genes within the RSAT package. Building on this concept, Bailey and Machanick (2012) implemented the CentriMo tool as part of the MEME suite. By comparing the distributions of DistMCPs for a given TF in the target and background sequences, we can identify those TFBSs that are positionally enriched relative to the MCP in the target set. As for Fisher analysis, KS scores are obtained by taking negative logarithms of the p-values obtained from KS tests.

#### False Discovery Rate calculation:

The False Discovery Rate (FDR) procedure was used to calculate adjusted p-values for both Z-scores and Fisher scores in Tables 2–5 (Benjamini and Hochberg 1995). Z-scores were first converted to p-values using the standard normal table, whereas for Fisher scores, the p-values obtained during Fisher exact probability calculation were used. All calculations were performed in the R statistics package (p.adjust for FDR and pnorm for the standard normal table). As mentioned in the original oPOSSUM article (Ho Sui *et al.* 2005), we do not perceive that TFBS over-representation measures are appropriate significance tests, as the procedures do not fully account for the non-random properties of genome sequences. The FDR values in Tables 2–5 were provided for completeness, but they do not affect the relative rankings.

### Single Site Analysis

Single Site Analysis (SSA) is the original over-representation analysis procedure developed for oPOSSUM. Individual TFBS hits within the user-specified search regions are counted for both the foreground gene/sequence set and the background set. For gene-based analysis, the search regions are determined by selecting the conserved regions located within a given distance from the gene transcription start sites. Both the Z-scores and Fisher scores are calculated for each TFBS based on the counts, which are then ranked accordingly. For a detailed explanation of the oPOSSUM SSA, please refer to Ho Sui *et al.* (2005). The major difference in the SSA implementation between the previous versions of oPOSSUM and version 3 is the replacement of the pairwise sequence alignment-based phylogenetic footprinting procedure with phastCons scores. Conserved regions are defined as those genomic regions of at least 20 nucleotides in length with average phastCons scores over a given threshold (two sets of conserved regions are determined using thresholds of 0.4 and 0.6), and TFBS searches are restricted to motifs within or overlapping (by at least 1 bp) these regions (Figure S3).

### Anchored Combination Site Analysis (aCSA)

In many cases, given a gene set of interest, the user has beforehand knowledge of a TF that is central to the observed co-regulation. To focus the intensive computational analysis of motif combinations, the aCSA method restricts the over-representation assessment to sequence regions proximal to predicted TFBS for a user-specified TF. Given an anchoring TF and an inter-binding site distance parameter value, pairings of the anchoring TFBS and all available secondary TFBSs within the inter-binding site distance are counted and over-representation scores are calculated. It is possible to observe self-interactions, when TFBSs for the anchor TF frequently occur in clusters.

### Gene-based *vs.* sequence-based

Increasingly researchers focus on sets of sequences likely to be bound by a TF, rather than a list of genes. To facilitate the analysis of sequence-based data, such as those from ChIP-Seq studies, new functions within oPOSSUM-3 were implemented. The sequence-based oPOSSUM systems take as input a foreground sequence set and a background sequence set. The supplied sequences are searched for TFBS motifs, and over-representation scores are calculated. There is no conservation filtering applied to the input sequences; the entire set of submitted sequences is screened for TFBS motifs.

### TFBS clustering

**Figure 2:**
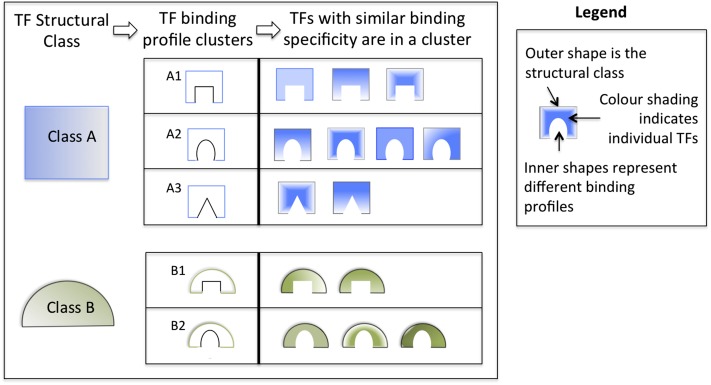
TF structural families and TFBS clusters. As many TFs cannot be distinguished in their binding specificity, clustering TF binding profiles promotes user consideration of all members of a functionally equivalent group. As TFs within a group bind to essentially identical sequences, users should focus on those TFs within a group likely to be active in a cell or condition relevant to their research. In the figure, the outer shapes and color represent the structural class, and the inner shapes symbolize the binding specificity of a cluster. The different shading within the shapes denotes individual TFs.

Transcription factors can be classified according to structural characteristics of DNA binding domains (Figure 2). It is often the case that TFs belonging to the same structural classes share similar DNA binding profiles, such that distinct TFs of the same class may bind to similar sequence patterns. If a given class contains numerous TFs with almost identical consensus sequences, oPOSSUM-3 analysis results can be dominated by subsets of profiles that are nearly identical. In such cases, it is useful to condense the redundant results to allow the user to identify independent enriched profiles. It is not suitable to combine the results simply based on the TF class, as the extent of the binding sequence similarity is variable among the different classes. Although some classes are defined by a characteristic consensus sequence, other classes, such as zinc fingers, have low profile similarities among the member TFs. Thus, it is necessary to divide each structural class into clusters based on profile similarity. Figure 2 illustrates the idea behind TFBS clustering and its application to oPOSSUM-3.

**Figure 3:**
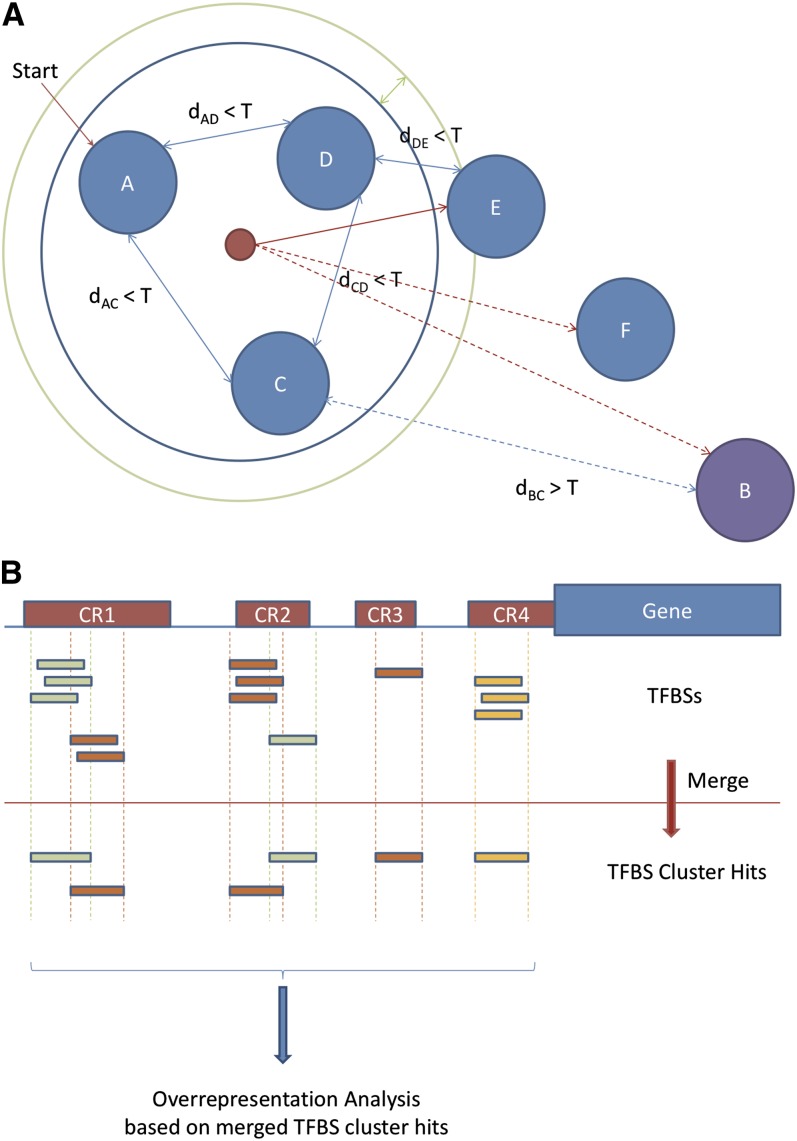
TFBS cluster analysis. (A) TFBS clustering process. Binding site profiles (represented by solid blue circles) are compared, and those exhibiting similarity (all pairwise similarity scores within a threshold T) are classified as a group (placed within the blue ring). In a second step, a consensus of the initial set is generated, and all additional profiles within the radius margin threshold (represented by the green ring) are added to the group. (B) Individual TFBS hits within a DNA sequence are grouped together according to the clustered groups, and over-representation analysis is performed from the group perspective. Each labeled red rectangle (*e.g.*, CR1) represents a cluster of TFBSs (individually displayed as small rectangles below).

Profiles in JASPAR 2010 have been annotated for TF structural class and family. Based on these classifications, the profiles in the given family are subject to a refined clustering process in oPOSSUM-3. First, using the MatrixAligner similarity scoring program (Sandelin *et al.* 2003), a pairwise similarity score table is calculated for the entire set of profiles in JASPAR. Two thresholds are set: (1) cluster score threshold T, which is the MatrixAligner score above which the two matrices being compared are deemed to be similar, and (2) radius margin R, which is a secondary score threshold used to determine whether those TFs at the boundary of the cluster join the cluster (Figure 3A). The process is based on tree traversal, with nodes being the profiles and the edges being the similarity scores. One profile within a given family is randomly chosen to act as the seed node for the cluster, and a tree is constructed between this seed profile and all other profiles in the family. The nodes are traversed in sequence, and the traversed nodes are added to the cluster if (1) the similarity scores between the parent node and the child node are lower than the cluster score threshold T, and (2) the average score S between the cluster member nodes (the parent nodes that have already been included in the cluster) and the child node is below T. If the child node in question qualifies for condition 1 but not condition 2, it can still be included in the cluster if S exceeds T by less than R. A pseudocode of the clustering process is given in Figure S4. From the JASPAR 2010 database, 250 profiles from the CORE collection and 184 from the PBM collection were analyzed, along with the 4 profiles from the custom PENDING collection. A cluster score threshold of 1.8 and a radius margin of 0.1 were used. These values were selected empirically, based on the distribution of pairwise similarity scores among all available JASPAR profiles.

When a TFBS cluster-based analysis is performed, any overlapping TFBS hits that belong to the same TFBS cluster are merged to form a single cluster hit. Only the merged cluster hits are counted for TFBS over-representation calculations (Figure 3B).

### Species-specific implementation details

The amount of sequence analyzed for the pre-computation of putative TFBS locations for the gene-based over-representation variants was adjusted to reflect the intergenic distances (unpublished observations) of the target organism. For human and mouse, 10,000 bp upstream and 10,000 bp downstream from the Ensembl-annotated TSS were searched for TFBS hits. For fruit fly, 3000 bp upstream and 3000 bp downstream were searched, and for nematode, 1500 bp in each direction. For yeast, 1000 bp upstream of TSSs and downstream to the 3′ end of each gene were searched. In gene-based pre-calculations for invertebrates, exons were excluded, as in general they are highly conserved and thus the conservation filter employed to reduce false positive TFBSs is rendered ineffective. As opposed to vertebrates and insects, nematodes exhibit some operon structures of gene organization (Blumenthal and Gleason 2003). An operon consists of multiple adjacent genes that are transcribed as a single unit, which is then spliced into separate mRNAs for translation. When analyzing genes that are part of an annotated operon, oPOSSUM-3 accounts for the operon structures by restricting the search space to the regions flanking the annotated start position of the first gene in the operon. TFBS predictions in the search region of the first gene are deemed to apply to all other genes in the operon (Figure S5).

Although JASPAR now includes distinct divisions for nematode and insect TFBS profiles, coverage is not optimal. For insects, the homeodomain family heavily dominates the set of profiles (90 of 125). For worms, there are currently only 4 profiles in the database. Thus, whereas human and mouse oPOSSUM-3 databases are built with vertebrate profiles only, fruit fly and nematode versions are built with all available metazoan profiles in JASPAR 2010.

The yeast gene-based oPOSSUM-3 database differs from the metazoan versions of oPOSSUM-3 in that it does not use conservation filtering, reflecting the compact promoter regions analyzed.

### Build process for oPOSSUM-3

**Figure 4:**
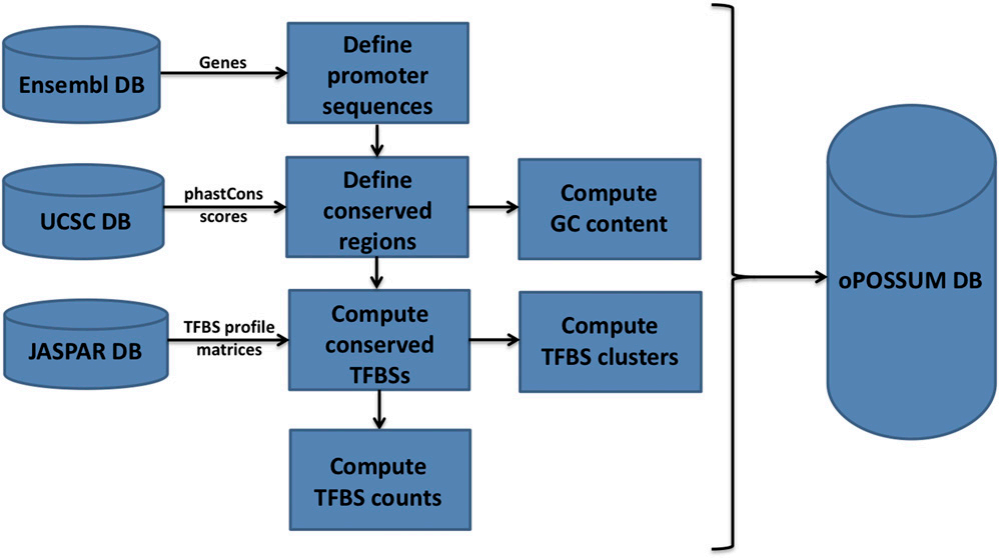
The build process for oPOSSUM 3 gene-based analysis. The system incorporates data from the gene annotation data from Ensembl, TFBS profiles from JASPAR, and multi-species conservation information based on phastCons scores from UCSC Genome Browser. The oPOSSUM database incorporates the data from these sources to pre-compute TFBS profile hits for gene-based analysis.

Figure 4 outlines the build process for gene-based versions of oPOSSUM-3. The system imports the gene and transcript annotation data from the Ensembl database and the phastCons conservation scores from the UCSC database. These data are combined to define the conserved segments in each gene. TFBS profiles from the JASPAR database are used to search for putative TFBSs within or overlapping the conserved regions. These TFBSs are stored in the oPOSSUM-3 database as well as the resulting counts (both single site and cluster sites) for pre-determined conservation thresholds, search region lengths, and profile matrix scoring thresholds. This pre-computation facilitates faster analysis for the user if these pre-determined search regions and thresholds are used for analysis. If the user chooses custom values of these search region lengths and thresholds, TFBS counts are computed at the time of the analysis. The sequence-based oPOSSUM-3 foregoes this pre-computation pipeline, and all calculations are done at the time of analysis.

### Data sources for ChIP-Seq–based analyses

ChIP-Seq datasets for Nfe2L2 (mouse embryonic fibroblast) and FoxA2 (mouse liver) were obtained in their final processed form from their respective authors (Malhotra *et al.* 2010; Robertson *et al.* 2008). The cMyc and Sox2 ChIP-Seq datasets [mouse embryonic stem cells (mES)] (Chen *et al.* 2008) were obtained as BED files from Gene Expression Omnibus (GEO accession GSE11431) (Edgar *et al.* 2002) and processed into peaks (putative TF bound regions) using the FindPeaks algorithm (Fejes *et al.* 2008) with the following settings: -control -dist_type 1 200 -subpeaks 0.6 -trim 0.2 -duplicatefilter. All NCBI36/mm8 datasets were converted to build NCBI37/mm9 using the UCSC Utility: Batch Coordinate Conversion (liftOver).

### ChIP-Seq–related analysis

Data analyses and plots were derived using the R statistical package (R Development Core Team 2008).

#### Selection of foreground datasets:

The FoxA2-, cMyc-, and Sox2-bound regions for our analyses were identified using a ratio of peak height to width, and the top 1200 ranked regions were selected. Nfe2L2 had undergone stringent filtering by the authors and had a strong signal in the data; thus the full dataset of 1256 sequences was used. For consistency, we chose similarly sized datasets for our analyses because the over-representation scores are dependent upon the data size (Figure S6).

#### Selection of background datasets:

Background datasets were extracted from the control data of the related ChIP-Seq experiments. All backgrounds were selected to match the mononucleotide GC composition of the foreground, except where we tested the impact of using different background GC compositions. The default background size for our analyses was 2-fold greater than the matching foreground, except for Nfe2L2 where the fibroblast background was limited to the same size as the foreground to obtain sequences with GC composition distribution similar to the foreground. Data analyses and plots were derived using the R statistical package (R Development Core Team 2008).

#### Thresholding by score:

The ranges of the Z-score and Fisher score enrichment values are dependent on foreground dataset size (see Figure S6). A threshold was derived using the mean plus N times the standard deviation of the score of interest (where N = 2 for Z-score thresholds, and N = 1 for Fisher score thresholds) (see Figure S7). KS scores can be undefined (infinite) for TFs of interest; for depiction, a score of 100 is assigned. Calculation of candidate thresholds excludes these values (Figure S8). Hereafter, the threshold is referred to as the applied threshold. In cases where we assess the correlation between reported results, we used lists of length 5 through M, where M was calculated to be four times the number of TFs with scores greater than the applied threshold. We chose to make M lenient in terms of the number of TFs that are enriched to be thorough.

#### GC composition calculation:

Sequence composition of ChIP-Seq sequences was determined at the mononucleotide level, using counts of A, G, C, and T. TF profile composition was determined similarly by counting the frequency of the nucleotides in all the sequences contributing to the profile. All backgrounds used in testing were selected to reflect the sequence GC composition distribution of the foreground dataset.

### Analysis parameters

All analyses were performed with the default parameters, unless otherwise stated. Default settings restrict oPOSSUM-3 results to those profiles with information content of at least 8 bits, and to putative TFBSs whose score is at least 85% of the optimal score. For gene-based analyses, species-specific default search region distances were used (corresponding to Level 3 in Table 1).

**Table 1:** Search region level distances for human/mouse, fly and nematode in oPOSSUM-3. oPOSSUM-3 searches for the presence of motifs in non-coding regions meeting a specified conservation threshold and situated within a selected search region.

	Human, Mouse		Fruit Fly		Nematode	
Search Region Level	Upstream (bp)	Downstream (bp)	Upstream (bp)	Downstream (bp)	Upstream (bp)	Downstream (bp)
1	10,000	10,000	3000	3000	1500	1500
2	10,000	5000	3000	2000	1000	1000
3	5000	5000	2000	2000	1000	500
4	5000	2000	2000	1000	500	500
5	2000	2000	1000	1000	250	250
6	2000	0	1000	0	250	0

## System Walk-Through

**Figure 5:**
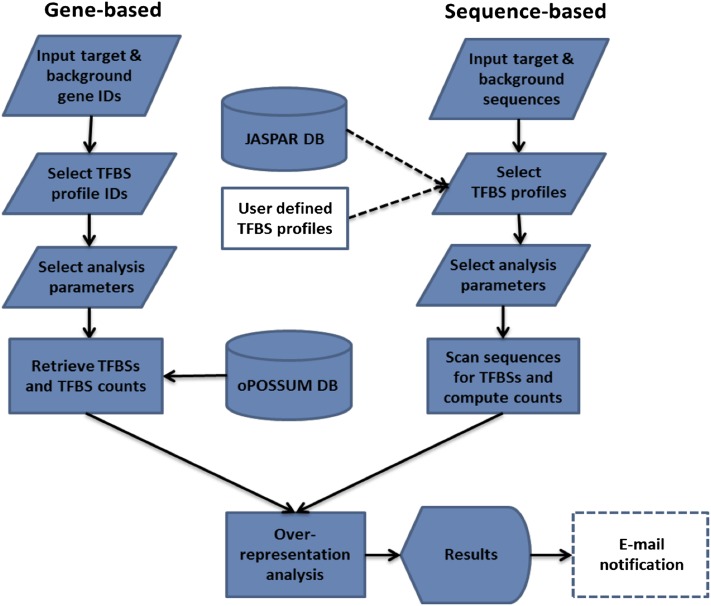
oPOSSUM analysis pipeline. Dashed lines indicate optional stages.

**Figure 6:**
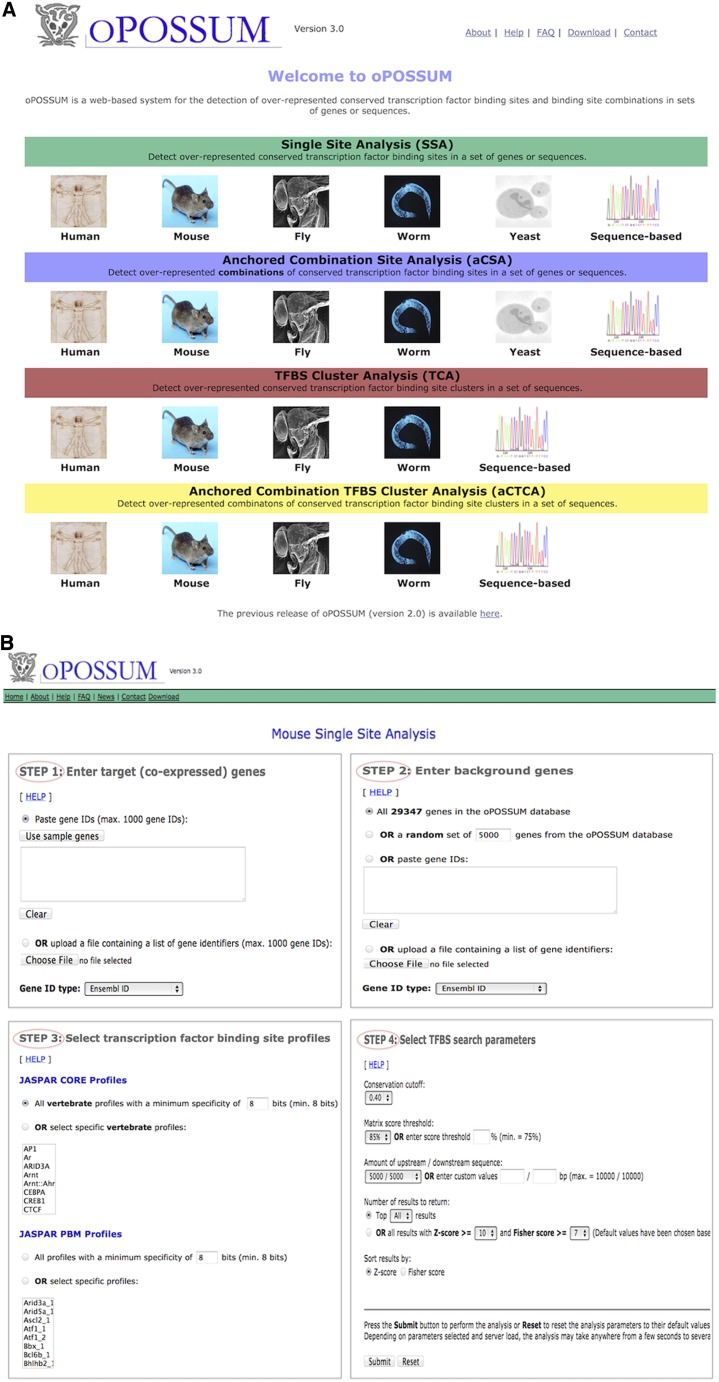
Web interface. (A) oPOSSUM home page listing the various analyses and organisms available. (B) Four steps for initiating a Single Site Analysis (SSA) with user-chosen parameters.

oPOSSUM-3 is available as a web service at http://opossum.cisreg.ca. The main page of oPOSSUM-3 lists each of the analysis methods offered as well as the species supported, allowing the user to select the appropriate combination. Figure 5 depicts the oPOSSUM-3 analysis pipeline for both the gene-based and sequence-based variants, and Figure 6 depicts the user interface.

### Input for gene-based analysis

For a gene-based analysis, the system takes as input the list of identifiers (ID) of the genes to be analyzed. Although the default input ID type is an Ensembl gene ID, the system accepts nine alternatives, including official gene symbols and Uniprot IDs. For the background gene set, the user can (1) choose the entire gene set from the oPOSSUM-3 database, (2) specify a number of genes to be randomly chosen, or (3) supply a list of gene IDs.

The gene-based analysis can be performed in either default or custom modes. In default mode, the user is restricted to pre-defined search regions, sequence conservation levels, and TFBS score thresholds. While in custom mode, the user can specify the parameter values within ranges allowed by the system. The default mode is faster as it makes use of pre-computed TFBS counts from the database, whereas the custom mode requires the system to compile binding site frequencies. For SSA, the user may select profiles from species-dependent subsets of JASPAR CORE, JASPAR PBM, or oPOSSUM-3-specific PENDING collections. The PENDING collection contains profiles that are not available from the 2010 release of JASPAR. The user specifies whether to use the entire set or a selected subset of the profiles. For aCSA, the user must select an anchor profile. For the TFBS cluster-based versions (both TCA and aCTCA), instead of selecting individual TFBS profiles, the user selects the TF of interest. The system will then include all TFBS clusters that belong to the selected TF families.

### Input for sequence-based analysis

The input parameter selection for the sequence-based methods is simpler than it is for the gene-based methods, as there are no search region or conservation level settings to specify. The user must supply fasta formatted sequences for both foreground and background datasets to be scanned for TFBS hits. oPOSSUM-3 provides a link to the Galaxy service for users needing to generate fasta files from sequence coordinates (Goecks *et al.* 2010). oPOSSUM-3 provides background sets for users lacking a matched background for their data. Lastly, the user must select TFBS profiles to be included in the analysis or provide a set of custom profiles.

### Parameter considerations

For all analyses, we suggest using default parameters, as we have done in this article, to get an analysis started when a user is uncertain of what to expect from their data. After the default analysis, users may elect to alter parameters for further investigation. It can be informative to compare how results change as parameters are altered. For instance, a score of 85% is the default threshold for reporting TFBSs; we have found this setting to work well with our datasets. Users may start with this threshold and decide that the results returned are missing a known TFBS, and so on the next analyses, they might reduce the TFBS stringency from 85 to 80%. Another example would be the region to analyze for gene-based analyses. The default distances are 5000 bp both up- and downstream; however, users may be interested to determine whether there are TFBSs enriched predominantly in upstream regions that differ from TFBSs enriched in downstream regions. In this case, two separate analyses would be run with different restrictions on the search regions.

### Results output

The results are generated and returned in a table format, along with a summary of the user-specified parameters and the nucleotide compositions of the foreground and background sequences used in the analysis. In gene-based SSA, the results table is generated and returned to the user immediately, whereas for gene-based aCSA, TCA, and aCTCA, as well as for all sequence-based methods, a link to the results is emailed to the user after computations are complete. If needed, the user can download the results as a tab-delimited text file for further analysis or record keeping. By default, the results in the table are ranked by descending Z-score, and the user can specify the number of motifs to report based on one score (default is all results). The table can be re-sorted by any column by clicking on the column header. The TF names are linked to the corresponding entries from the JASPAR web site. As a profile’s information content (IC) can affect its rate of occurrence, those profiles with IC lower than 9 or greater than 19 are highlighted to inform the user of extreme cases. The TFBS counts are linked to a separate TFBS details page that specifies the sequences and locations. For sequence-based analysis, the report specifies the overall GC mononucleotide content for both the background and foreground sequence sets. For both gene-based and sequence-based SSA and aCSA, the results table reports the GC content of each TF binding profile and highlights the extreme values (*i.e.*, those that are below 0.33 or above 0.66). For TFBS cluster-based versions, the results table entries are based on TFBS cluster names instead of individual profiles. The TFBS cluster hit coordinates and the corresponding sequences represent the merged TFBS hits. Each TFBS cluster name is linked to a separate page with the summary information on the cluster, such as TF class, family, and the member profiles that constitute the cluster.

**Figure 7:**
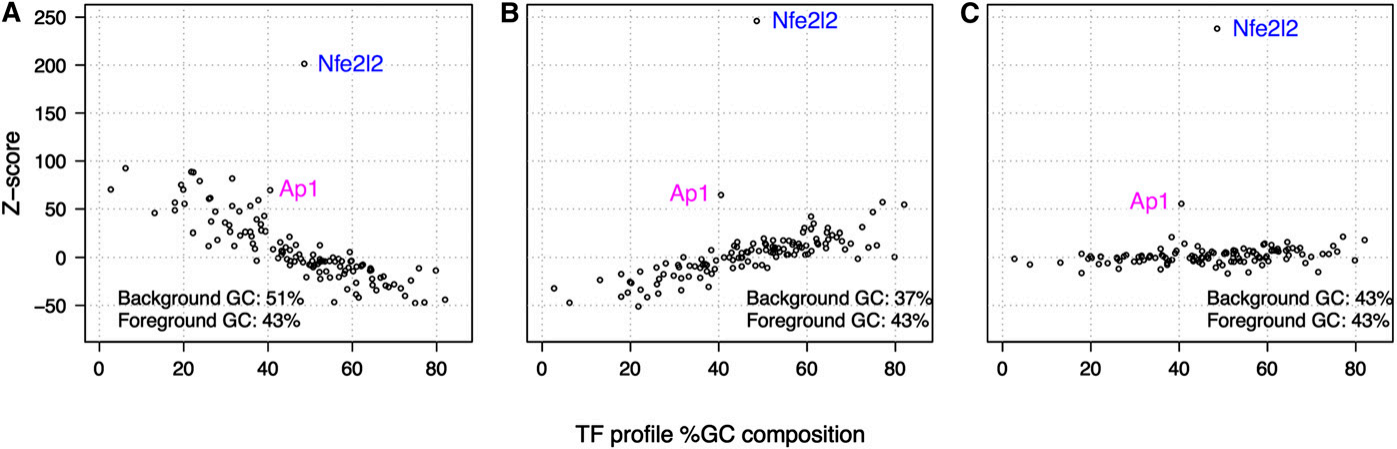
Relationship between TF profile GC content and enrichment statistics. The percentage of G and C nucleotides in the TF profile models are plotted against the motif enrichment Z-scores. The three panels represent analysis results for the same 1256 Nfe2L2 ChIP-Seq regions (GC composition avg. 43%) compared with three background sets of different GC composition: (A) elevated background GC (avg. 51% GC); (B) low background GC (avg. 37% GC); and (C) background with GC composition matched to the distribution of the ChIP-Seq regions (avg. 43% GC). The GC composition of the background used in B is that of the control associated with the Nfe2L2 ChIP-Seq data. The plotted Z-scores represent the enrichment of TFs in 1256 ChIP-Seq Nfe2L2-bound regions. The Nfe2L2 profile can be distinguished in all three cases (A, B, and C). However, detection of the Ap1 profile, representing a TF with a known Nfe2L2-related biological function, is sensitive to background selection. Most TFs that would be ranked highly by Z-score when the background is not corrected for GC composition (seen in A and B) are not ranked highly when the background is matched to the foreground distribution of GC composition (C).

Regarding interpretation of the results, we strongly recommend that users consider results plotted as we have done in this article [*i.e.*, in Figure 7 and Figure S8, the score of interest (Z-score, Fisher score, or KS score) *vs.* the GC composition of the TF profile (only for SSA and aCSA), and in Figure S7, the Fisher score *vs.* Z-score]. This visualization of the data will alert the user if a skew in TF ranking has occurred due to imbalanced composition of the background relative to the foreground. If the user wants an alternative computable metric, we recommend as a first pass setting a score threshold using the median plus N times the standard deviation (see *Materials and Methods*). For SSA and TCA, start with N = 2 for Z-score, and N = 1 for Fisher score; for aCSA or aTCA, start with N = 2 or 3 for either score. It is useful to consider these thresholds in conjunction with the aforementioned plots as a means for deciding whether the threshold is too stringent for the user’s purposes.

## Results

### Application to reference collections

To validate the performance of the oPOSSUM-3 system, each of the analysis methods was tested using either sets of co-expressed genes or sequences identified in ChIP-Seq studies. The gene sets were restricted to cases in which there was prior knowledge of TF(s) responsible for co-expression. The ChIP-Seq sequences, by the nature of the ChIP method, are already self-restricted to a primary TF (the target of the antibody used in the experiments).

### Skeletal muscle reference gene set

Skeletal muscle-specific genes are known to be regulated by a core set of TFs including (but not limited to) Mef2A, Myf, Srf, Tead1, and Sp1, with the first three performing prominent roles (Braun *et al.* 1994; Naya and Olson 1999; Rudnicki and Jaenisch 1995; Wasserman and Fickett 1998). From the literature, a collection of 25 human genes regulated by muscle-specific enhancers was prepared (Table S1). This set was analyzed with the full panel of oPOSSUM-3 gene-based methods for human, and the results are shown in Table 2. Although rankings differ depending on the type of analysis performed and the scoring method used, one or more of the core muscle-specific TFs of Mef2A, Myf, and Srf are included in the top five enriched TF profiles by either the Z- or Fisher scores. SSA (Table 2A) is able to identify the muscle factors Mef2A, Myf, Srf, and Sp1 as candidate TFs for regulating the muscle gene set, along with Nkx2-5, which is also a known muscle-specific TF (Durocher *et al.* 1997). Likewise, TCA (Table 2B) yields the clusters C113 (Nkx2-5), C130 (Mef2A), C143 (Myf), and C129 (Srf). When run with Mef2A as the anchoring TF, aCSA (Table 2C) reports known muscle regulatory TF candidate pairings with Mef2A, including Sp1, Nkx2-5, and Klf4 (Yoshida *et al.* 2010). Similarly for aCTCA (Table 2D), Mef2A pairs with ETS domain TFs (a known muscle regulatory TF family) in addition to clusters containing Sp1 and Klf4.

**Table 2:** oPOSSUM-3 results for the muscle reference gene set (human).

**A. Single Site Analysis using JASPAR CORE vertebrate profiles. Nkx2-5 is a known muscle regulatory TF involved in cardiac muscle development. The bHLH TFs Nhlh1 and Myf have similar binding profiles.**				
Z-score	Name	Class:Family	Score	FDR
	**Nkx2-5**	**HTH:Homeo**	**31.4**	**2.85E-215**
	Arid3A	HTH:Arid	29.3	6.26E-187
	**Mef2A**	**Other Alpha-Helix:MADS**	**28.0**	**1.79E-171**
	HoxA5	HTH:Homeo	27.1	4.97E-160
	Pdx1	HTH:Homeo	26.5	1.28E-153
Fisher score	**Mef2A**	**Other Alpha-Helix:MADS**	**23.6**	**6.53E-09**
	**Myf**	**Zipper-type:HLH**	**15.5**	**1.08E-05**
	**Sp1**	**Zinc-coord:ZnF**	**13.6**	**3.60E-05**
	**Pparg::Rxra**	**Zinc-coord:NucReceptor**	**12.9**	**3.60E-05**
	**Srf**	**Other Alpha-Helix:MADS**	**12.0**	**5.80E-05**
**B. TFBS Cluster Analysis. TFs of interest are shown in parentheses under the cluster name.**				
Z-score	Name	Class:Family	Score	FDR
	**C113 (Nkx2-5)**	**HTH:Homeo**	**49.4**	**0.00E+00**
	C1 (TATA)	Beta-sheet:TATA-binding	35.6	2.25E-275
	C14 (Arid3A)	Winged HTH:Arid	31.9	8.47E-222
	C57 (FoxA1)	Winged HTH:Forkhead	31.9	1.55E-221
	**C130 (Mef2A)**	**Other Alpha-helix:MADS**	**29.1**	**1.46E-185**
Fisher score	**C130 (Mef2A)**	**Other Alpha-helix:MADS**	**23.7**	**8.66E-09**
	**C19 (Pparg:Rxra)**	**Zinc-coord:NucReceptor**	**16.3**	**7.09E-06**
	**C143 (Myf)**	**Zipper-type:HLH**	**15.2**	**1.42E-05**
	**C129 (Srf)**	**Other Alpha-helix:MADS**	**12.7**	**1.30E-04**
	C108 (Myb)	HTH:Myb	11.7	2.60E-04
**C. Anchored Combination Site Analysis. (Anchor: Mef2A, Maximum Inter-Binding Distance: 100 bp)**				
Z-score	Name	Class:Family	Score	FDR
	**Sp1**	**Zinc-coord:ZnF**	**37.4**	**1.05E-303**
	**Nkx2-5**	**HTH:Homeo**	**34.2**	**5.70E-255**
	**Gata1**	**Zinc-coord:Gata**	**32.9**	**2.05E-236**
	**Pparg::Rxra**	**Zinc-coord:NucReceptor**	**29.8**	**1.86E-193**
	YY1	Zinc-coord:ZnF	29.2	7.63E-186
Fisher score	**Sp1**	**Zinc-coord:ZnF**	**36.9**	**1.09E-14**
	Znf354C	Zinc-coord:ZnF	22.1	1.32E-08
	**Klf4**	**Zinc-coord:ZnF**	**21.7**	**1.32E-08**
	Mzf1_1-4	Zinc-coord:ZnF	21.5	1.33E-08
	Zeb1	Zinc-coord:ZnF	16.0	2.61E-06
**D. Anchored Combination TFBS Cluster Analysis. (Anchor: Mef2A, Maximum Inter-Binding Distance: 100 bp)**				
Z-score	Name	Class:Family	Score	FDR
	**C113 (Nkx2-5)**	**HTH:Homeo**	**47.0**	**0.00E+00**
	**C75 (Sp1)**	**Zinc-coord:ZnF**	**41.9**	**0.00E+00**
	**C55 (Ets1, Elk1/4)**	**Winged HTH:Ets**	**34.2**	**7.55E-255**
	**C19 (Pparg::Rxra)**	**Zinc-coord:NucReceptor**	**32.0.3**	**1.20E-227**
	**C2 (Gata1)**	**Zinc-coord:GATA**	**31.3**	**5.14E-214**
Fisher score	**C104 (Klf4)**	**Zinc-coord:ZnF**	**22.9**	**1.93E-08**
	C66 (Znf354C)	Zinc-coord:ZnF	22.2	1.94E-08
	**C75 (Sp1)**	**Zinc-coord:ZnF**	**21.7**	**2.13E-08**
	**C19 (Pparg::Rxra)**	**Zinc-coord:ZnF**	**19.1**	**2.15E-07**
	C72 (Zeb1)	Zinc-coord:ZnF	17.8	6.32E-07

Known muscle TFs are bolded. FDR (false discovery rate) column lists the adjusted p-values calculated using the Benjamini & Hochberg algorithm. (Background: 5000 random, Conservation: 0.4, Matrix Score Threshold: 85%, Search region: ± 5000 bp.)

### Cilia gene set for nematodes

Inglis *et al.* (2006) curated a collection of genes known to be involved in cilia function and structure, making the set available within Ciliome DB. In vertebrates, RFX TFs are the key regulators of cilia gene expression. The RFX TFs bind to a regulatory motif termed the X-box. The regulation is conserved between mammals and nematodes; X-box sequences are associated with orthologous cilia genes (Efimenko *et al.* 2005). Although a nematode-specific RFX motif has been computed from these sequences, these sequences were not experimentally validated in a rigorous manner. There are two RFX profiles in the research literature, differing in the distance between two half sites (Emery *et al.* 1996). These RFX profiles were added to the oPOSSUM-3 custom collection of TFBS profiles and were used to analyze 531 nematode cilia genes. The gene set was obtained from Ciliome DB by combining the nematode genes from Avidor-Reiss, Blacque SAGE, and Efimenko datasets and selecting for those genes that were included in at least two of the studies. The results are listed in Table 3. RFX profiles received the highest scores in both SSA and TCA. The high scores obtained using the vertebrate RFX profiles in nematode genomic sequences support the conservation of the TFBS sequences across large evolutionary distances.

**Table 3:** oPOSSUM-3 results for the cilia gene set in nematodes.

**A. Single Site Analysis using JASPAR CORE collection and the custom PENDING collection. Two variants of Rfx1 profiles are from TRANSFAC (Rfx1_1 and Rfx1_2), which were placed into the custom PENDING collection for analysis.**				
Z-score	Name	Class:Family	Score	FDR
	**Rfx1_2**	**HTH:RFX**	**55.8**	**0.00E+00**
	**Rfx1_1**	**HTH:RFX**	**35.7**	**1.90E-277**
	Spib	Winged HTH:Ets	8.8	2.75E-17
	Esr2	Zinc-coord:NucReceptor	8.7	5.61E-17
	Arnt::Ahr	Zipper-type:HLH	7.4	1.88E-12
Fisher score	**Rfx1_2**	**HTH:RFX**	**36.6**	**8.15E-24**
	Sox5	Other Alpha-helix:HMG	30.1	8.02E-15
	HoxA5	HTH:Homeo	29.5	3.56E-12
	Spib	Winged HTH:Ets	29.2	4.86E-12
	Sox17	Other Alpha-helix:HMG	28.6	5.25E-12
**B. TFBS Cluster Analysis. C154 contains RFX profiles from TRANSFAC, and C155 consists of RFX profiles from the JASPAR PBM collection.**				
Z-score	Name	Class:Family	Score	FDR
	**C154 (Rfx1)**	**HTH:RFX**	**30.9**	**2.37E-208**
	**C155 (Rfx3,4)**	**HTH:RFX**	**25.0**	**1.02E-136**
	C62	Winged HTH:E2F	12.5	2.66E-34
	C39	Zinc-coord:ZnF	10.7	1.41E-25
	C127	Winged HTH:Ets	8.5	2.49E-16
Fisher score	**C154 (Rfx1)**	**HTH:RFX**	**38.4**	**3.58E-15**
	C41 (Sox2)	Other Alpha-helix:HMG	34.8	6.55E-14
	C113 (Nkx2-5)	HTH:Homeo	34.0	9.71E-14
	**C155 (Rfx3,4)**	**Winged HTH:RFX**	**33.2**	**1.62E-13**
	C55 (Ets1, Elk1/4)	Winged HTH:Ets	32.6	2.36E-13

TFs known to be involved in cilia gene regulation are bolded. The scores for daf-19 profile are not shown, as the profile was built based on gene sets included in these analyses, making the results circular. FDR (false discovery rate) column lists the adjusted p-values calculated using the Benjamini & Hochberg algorithm. (Matrix Score Threshold: 85%, JASPAR CORE vertebrate profiles and JASPAR PENDING profiles, minimum information content = 8 bits.)

### Results for ChIP-Seq reference datasets

We used oPOSSUM-3 to analyze ChIP-Seq datasets, of which we describe two results here. The Nfe2L2 data from Malhotra *et al.* (2010) contains relatively few ChIP-Seq regions (1256 sequences) derived from studies of mouse embryonic fibroblasts. Nfe2L2, previously known as Nrf2, is a stress-activated TF linked to the regulation of detoxification enzymes. We analyzed the Nfe2L2 data with oPOSSUM-3 (Table 4). The Nfe2L2 profile is consistently found to be a top-scoring motif in all analyses. The Ap1 motif, known to overlap and coordinate with the Nfe2L2 binding sequence (Friling *et al.* 1992), was also enriched in the SSA results, and it was clustered with the Nfe2L2 profile (cluster ^11^C) in the TCA and aCTCA analyses.

**Table 4:** oPOSSUM-3 results for Nfe2L2 ChIP-Seq dataset, using JASPAR CORE vertebrate profiles.

**A. Single Site Analysis. Nfe2L2 ranks first and Ap1 sec by all three scores.**				
Z-score	Name	Class:Family	Score	FDR
	**Nfe2L2**	**Zipper-Type:LeuZip**	**238.0**	**0.00E+00**
	**Ap1**	**Zipper-Type:LeuZip**	**55.5**	0.00E+00
	Klf4	Zinc-coord:ZnF	21.6	2.60E-102
	Irf1	Winged HTH:Irf	21,2	2.01E-98
	Sp1	Zinc-coord:ZnF	18.2	1.17E-72
Fisher score	**Nfe2L2**	**Zipper-Type:LeuZip**	**457.8**	**1.73E-197**
	**Ap1**	**Zipper-Type:LeuZip**	**28.9**	1.68E-11
	Irf1	Winged HTH:Irf	9.6	2.50E-03
	Klf4	Zinc-coord:ZnF	6.3	5.56E-02
	Sp1	Zinc-coord:ZnF	5.3	1.20E-01
KS score	**Nfe2L2**	**Zipper-Type:LeuZip**	**Inf**	**0.00**
	**Ap1**	**Zipper-Type:LeuZip**	**Inf**	0.00
	Nkx2-5	HTH:Homeo	28.8	1.20E-11
	HoxA5	HTH:Homeo	28.2	1.66E-11
	Arid3A	HTH:Arid	26.7	5.95E-11
**B. TFBS Cluster Analysis. Nfe2L2 and Ap1 are both in cluster ^11^C.**				
Z-score	Name	Class:Family	Score	FDR
	**^11^C (Nfe2L2, Ap1)**	**Zipper-type:LeuZip**	**118.8**	**0.00E+00**
	C4	Winged HTH:IRF	23.3	1.55E-118
	C104	Zinc-coord:ZnF	21.7	8.83E-104
	C108	HTH:Myb	13.7	2.85E-42
	C107	Other:Cp2	13.1	2.96E-38
Fisher score	**^11^C (Nfe2L2, Ap1)**	**Zipper-type:LeuZip**	**35.3**	**8.17E-14**
	C4	Winged HTH:IRF	10.3	2.95E-03
	C104	Zinc-coord:ZnF	6.3	1.09E-01
	C107	Other:Cp2	4.9	2.62E-01
	C108	HTH:Myb	4.9	2.62E-01
KS score	**^11^C (Nfe2L2, Ap1)**	**Zipper-type:LeuZip**	**Inf**	**0.00**
	C55	Winged HTH::Ets	Inf	0.00
	C113	HTH:Homeo	Inf	0.00
	C41	Other Alpha-helix:HMG	Inf	0.00
	C57	Winged HTH::Forkhead	Inf	0.00
**C. Anchored Combination Site Analysis. Nfe2L2 associates most frequently with itself and Ap1. (Anchor: Nfe2L2, Maximum inter-binding distance = 100 bp)**				
Z-score	Name	Class:Family	Score	FDR
	**Nfe2L2**	**Zipper-Type:LeuZip**	**607.1**	**0.00**
	**Ap1**	**Zipper-Type:LeuZip**	**432.9**	**0.00**
	Klf4	Zinc-coord:ZnF	265.5	0.00
	Tcfcp2l1	Other:Cp2	260.0	0.00
	Znf354c	Zinc-coord:ZnF	251.8	0.00
Fisher score	**Ap1**	**Zipper-Type:LeuZip**	**391.0**	**1.17E-168**
	Znf354C	Zinc-coord:Znf	380.0	5.03E-164
	Sp1b	Winged HTH:Ets	340.0	7.40E-147
	Hoxa5	HTH:Homeo	336.0	3.93E-145
	Mzf1_1-4	Zinc-coord:ZnF	299.0	4.15E-129
**D. Anchored Combination TFBS Cluster Analysis. (Anchor: Nfe2L2, Maximum inter-binding distance = 100 bp)**				
Z-score	Name	Class:Family	Score	FDR
	**^11^C (Nfe2L2, Ap1)**	**Zipper-Type:LeuZip**	**193.8**	**0.00**
	C55 (Ets, Elk1)	Winged HTH:Ets	130.6	0.00
	C113	HTH:Homeo	109.8	0.00
	C104	inc-coord:ZnF	93.1	0.00
	C75	Zinc-coord:ZnF	92.2	0.00
Fisher score	**^11^C (Nfe2L2, Ap1)**	**Zipper-Type:LeuZip**	**88.1**	**9.31E-37**
	C55 (Ets, Elk1)	Winged HTH:Ets	40.9	1.47E-16
	C113	HTH:Homeo	34.8	4.36E-14
	C66	Zinc-coord:ZnF	32.8	2.42E-13
	C104	Zinc-coord:ZnF	28.9	9.55E-12

Nfe2L2 and Ap1, a TF with known Nfe2L2-related biological functions, are bolded. FDR (false discovery rate) column lists the adjusted p-values calculated using the Benjamini & Hochberg algorithm. (Matrix Score Threshold: 85%, JASPAR CORE vertebrate profiles, minimum information content = 8 bits.)

For contrast, the FoxA2 data from Robertson *et al.* (2008) shows a large number of TF bound regions (>10,000 sequences) using mouse liver as the source. We took a subset of 1200 sequences for analysis (see *Materials and Methods*). FoxA2 (also known as Hnf3α), a TF linked to differentiation, is a member of the forkhead-box family of TF proteins for which the JASPAR 2010 database has eight profiles. Using the sequence-based SSA, we recovered FoxA2 as the top ranking TF, while additional forkhead-box family profiles were ranked in the top five enriched TFBSs (Table 5). The other prominently enriched profile represents the Hnf4 hepatocyte nuclear factor, which is predominantly expressed in the liver. The TCA analysis complements the SSA results, with the clusters C57 representing forkhead-box binding profiles ranking first in all three scores. C19 (Hnf4A) ranks second by Z and Fisher scores, whereas C41 (Sox2, SRY) ranks second by KS score. aCSA and aCTCA analyses also support the importance of forkhead-box binding and Hnf4 profiles.

**Table 5:** oPOSSUM-3 results for FoxA2 ChIP-Seq dataset, using JASPAR CORE vertebrate profiles.

**A. Single Site Analysis Results. Forkhead-box binding profiles rank the highest by both Z-score and Fisher score.**				
Z-score	Name	Class:Family	Score	FDR
	**FoxA2**	**Winged HTH:Forkhead**	**203.4**	**0.00**
	FoxA1	Winged HTH:Forkhead	155.6	0.00
	FoxF2	Winged HTH:Forkhead	113.8	0.00
	FoxD1	Winged HTH:Forkhead	107.1	0.00
	FoxO3	Winged HTH:Forkhead	90.1	0.00
Fisher score	**FoxA2**	**Winged HTH:Forkhead**	**388.7**	**1.86E-167**
	FoxA1	Winged HTH:Forkhead	219.0	4.62E-94
	FoxD1	Winged HTH:Forkhead	131.4	3.20E-56
	FoxF2	Winged HTH:Forkhead	82.4	4.97E-35
	FoxO3	Winged HTH:Forkhead	77.2	6.71E-33
KS score	**FoxA2**	**Winged HTH:Forkhead**	**Inf**	**0.00**
	FoxA1	Winged HTH:Forkhead	Inf	0.00
	FoxD1	Winged HTH:Forkhead	Inf	0.00
	FoxF2	Winged HTH:Forkhead	Inf	0.00
	FoxO3	Winged HTH:Forkhead	Inf	0.00
**B. TFBS Cluster Analysis. Cluster C57, containing forkhead-box binding profiles, ranks highest by all three scores. Cluster C19, containing the Hnf4A hepatocyte-related TF profile, ranks second by Z and Fisher scores, while cluster C41 containing the Sox profiles ranks the same as C57 by KS score.**				
Z-score	Name	Class:Family	Score	FDR
	**C57 (FoxA2)**	**Winged HTH:Forkhead**	**214.0**	**0.00E+00**
	C19 (Hnf4A)	Zinc-coord:NucReceptor	67.2	0.00E+00
	C9	Zipper-type:LeuZip	54.3	0.00E+00
	C41 (Sox2, Sry)	Other Alpha-helix:HMG	31.6	5.29E-218
	C17	Zinc-coord:NucReceptor	29.3	1.92E-187
Fisher score	**C57 (FoxA2)**	**Winged HTH:Forkhead**	**69.2**	**1.44E-28**
	C19 (Hnf4A)	Zinc-coord:NucReceptor	9.2	8.88E-03
	C9	Zipper-type:LeuZip	1.3	1.00E+00
	C41 (Sox2, Sry)	Other Alpha-helix:HMG	0.012	1.00E+00
	C17	Zinc-coord:NucReceptor	0.005	1.00E+00
KS score	**C57 (FoxA2)**	**Winged HTH:Forkhead**	**Inf**	**0.00**
	C41 (Sox2,Sry)	Other Alpha-helix:HMG	35.6	9.66E-15
	C113	HTH:Homeo	20.7	1.98E-08
	C19	Zinc-coord:NucReceptor	18.6	1.22E-07
	C17	Zinc-coord:NucReceptor	17.7	2.48E-07
**C. Anchored Combination Site Analysis. FoxA2 associates closely with other forkhead-box binding profiles, and the profile of Hnf4A, a hepatocyte-related TF. (Anchor: FoxA2, Maximum inter-binding distance = 100 bp)**				
Z-score	Name	Class:Family	Score	FDR
	FoxA1	Winged HTH:Forkhead	334.8	0.00
	FoxA2	Winged HTH:Forkhead	265.3	0.00
	Hnf4A	Zinc-coord:NucReceptor	247.1	0.00
	FoxD1	Winged HTH:Forkhead	238.7	0.00
	FoxO3	Winged HTH:Forkhead	213.4	0.00
Fisher score	FoxA2	Winged HTH:Forkhead	245.7	2.23E-105
	FoxA1	Winged HTH:Forkhead	238.0	2.56E-102
	Zeb1	Zinc-coord:ZnF	236.5	7.57E-102
	Znf354C	Zinc-coord:ZnF	223.0	4.14E-96
	FoxD1	Winged HTH:Forkhead	208.0	1.07E-89
**D. Anchored Combination TFBS Cluster Analysis. Cluster C19 containing the Hnf4A hepatocyte-related TF profile is ranked high by both scores. (Anchor: FoxA2, Maximum inter-binding distance = 100 bp)**				
Z-score	Name	Class:Family	Score	FDR
	C57 (FoxA2)	Winged HTH:Forkhead	189.3	0.00
	C19 (Hnf4A)	Zinc-coord:NucReceptor	180.1	0.00
	C17	Zinc-coord:NucReceptor	151.1	0.00
	C41	Other Alpha-helix:HMG	135.0	0.00
	C9	Zipper-type:LeuZip	133.5	0.00
Fisher score	C57 (FoxA2)	Winged HTH:Forkhead	72.6	5.02E-30
	C19 (Hnf4A)	Zinc-coord:NucReceptor	42.2	4.00E-17
	C55	WingedHTH:Homeo	31.5	1.18E-12
	C113	HTH:Homeo	26.5	1.32E-10
	C9	Zipper-type:LeuZipper	25.0	4.72E-10

FoxA2 is bolded. FDR (false discovery rate) column lists the adjusted p-values calculated using the Benjamini & Hochberg algorithm. (Matrix Score Threshold: 85%, JASPAR CORE vertebrate profiles, minimum information content = 8 bits.)

### Accounting for GC composition in over-representation analyses

The genomes are composed of regions that are distinct by the nature of their nucleotide composition. Promoters for housekeeping genes in particular are often found to be rich in G and C nucleotides or in CpG islands (Yamashita *et al.* 2005), whereas tissue-specific promoters are often found to be relatively more AT-rich (Roider *et al.* 2009). These broad composition differences are reflected among the sequences derived from ChIP experiments, such as ChIP-Seq. The %GC content of the published ChIP-Seq experiments we examined ranged from ∼30–57% GC (unpublished observations). The mean and variance of the %GC composition distribution differed between experiments for different TFs. The binding profiles of the TFs themselves also varied in GC content. Given a set of GC-rich sequences to analyze, TFs with GC-rich profiles will have a greater probability of having a motif present than AT-rich profiles. As over-representation analysis is designed to report TFBS predictions that exist to a greater extent in a set of chosen sequences than in a background set of sequences, we examined the effect of GC composition on motif enrichment scoring using backgrounds with varying GC content (*i.e.*, high, low, and neutral GC composition) against several ChIP-Seq datasets.

We used mouse-derived ChIP-Seq data for several TFs with varying GC composition in their profiles: Sox2 (ES cells), 36% GC; Nfe2L2 (fibroblast), 43% GC; FoxA2 (liver), 46% GC; and cMyc (ES cells), 61% GC. For each ChIP-Seq dataset, we analyzed a subset of the TF bound regions (foreground data) against background sequence sets with a variety of %GC distributions. The over-representation Z-score for each TF was then plotted against the %GC composition of the TF’s profile. As illustrated with Nfe2L2 in Figure 7, composition differences between the foreground and background sequences will affect the over-representation scores of TFs. When the background %GC composition is lower than that of the foreground sequences, the TFs with low GC profiles are reported as under-represented (low Z-score) in the foreground sequences relative to the background sequences, while the TFs with high GC profiles are over-represented (high Z-score) in the foreground sequences *vs.* the background sequences. The opposite trend occurs when the background %GC composition is higher than the foreground sequences; in this case the TFs with low GC profiles are reported as over-represented. A background with the same GC composition distribution (mean and variance) as the foreground dataset removes bias arising from GC composition differences and controls against TFBSs being unduly assigned high or low over-representation scores.

### Scoring measures for ChIP-Seq–based analyses

In combination, the Z-score and Fisher score inform the user whether enrichment is (1) due to a high frequency of TFBSs in some of the sequences and absence in others (Z-score high rank, Fisher score low rank; Figure S7A, TF: Ap1); (2) due to a majority of regions carrying a TFBS but at low frequency per sequence (Fisher score high rank, Z-score low rank; or (3) due to both types of representation being elevated (Z-score and Fisher score both rank high). For sequence-based analyses on ChIP-Seq data, we find that ordering by either score presents similar ranks. To measure the agreement between the Z- and Fisher scores, we took the top 10 motifs ranked by each of the two types of scores and counted the fraction of TF motifs that were common to both lists. We based our choice of 10 top motifs on our lenient applied threshold (see *Materials and Methods*). Using the results from SSA, there was 100% agreement within the top 10 motifs for cMyc, and 82% agreement within both Sox2 and Foxa2. The Chip-Seq target TF ranked in the top two enriched motifs for both measures. For the most stringent results, users may use both Z- and Fisher over-representation scores in combination when selecting motifs for further analysis (*i.e.*, motifs scoring above both scores’ applied thresholds, Figure S7).

Unique to sequence-based SSA and TCA, KS scores are available, complementing Z- and Fisher scores. For the Nfe2L2 and FoxA2 ChIP-Seq datasets analyzed in this study, the target TF binding profiles receive high KS scores, as well as high Z- and Fisher scores (Tables 4 and 5 and Figure S8). The KS scores obtained for these datasets were extreme; “infinity” values were returned for the target profiles. The accompanying false discovery rates support the utility of these scores.

### Assessing impact of background dataset size on ChIP-Seq–based analyses

For some foreground sets, extreme composition properties can make it difficult to obtain composition-matched background sets of comparable size from the associated control dataset, let alone a background with 2-fold or more sequences. To determine the impact of background set size on results, we assessed the impact of background-to-foreground ratios on results. Datasets from ChIP-Seq analysis of the Sox2 and FoxA2 TFs were used for the assessment. We first assessed the variability between two different background sets of the same size, selected from the pool of control sequences to match the composition of the same associated foreground dataset. SSA analysis was performed for each. The Spearman correlation between the top TFs, for lists of length 5 through M (where M is derived from the lenient threshold; see *Materials and Methods*), was ≥0.97 for both Sox2 and FoxA2 Z-scores, ≥0.93 for FoxA2 Fisher scores, and ≥0.86 for Sox2 Fisher scores. Thus the selection of background sequences, which are matched to the foreground composition, has a subtle influence on the rankings of enriched TFs. We then ran SSA analyses using 0.5-, 1-, 2-, 3-, and 4-fold background-to-foreground ratios. For this comparison of different sized backgrounds, we again used lists of length 5 through M. The Spearman correlation between any two backgrounds, regardless of size, was ≥0.89 for both the Z- and Fisher scores of FoxA2, whereas the correlation value for Sox2 was ≥0.90 for either score. Thus we conclude that using background datasets larger than the foreground is not critical as it does not strongly influence the rankings of enriched motifs.

## Discussion

The oPOSSUM-3 system enables researchers interested in the study of gene regulatory networks to identify TFs that may be acting in a biological context. Several key advances in the implementation of oPOSSUM-3 make the system suitable for analysis of emerging high-throughput data. Using phastCons multi-species conservation scores obviates the restrictive pairwise sequence alignment phylogenetic footprinting procedures of past releases, which greatly increases the number of genes represented within the system. This conversion to phastCons-based phylogenetic footprinting enables the construction of oPOSSUM services for any species represented in the Ensembl genome annotation database and the UCSC Genome Browser. The new anchored approach to combination site analysis (aCSA) allows for computationally tractable identification of interacting TFs. Motivated by the highly similar profiles for structurally related TFs emerging from large-scale studies, the clustering of highly similar binding site profiles allows a more focused report to users. Most importantly, the new sequence-based methods enable the oPOSSUM system to function as an analysis tool for large-scale ChIP-Seq sequence sets. Taken as a whole, oPOSSUM-3 is a powerful tool for biologists seeking insight into gene regulatory networks.

One of the key considerations in motif enrichment analysis of mammalian regulatory sequences is how to filter the sequences to enrich for regulatory regions. The presented methods have two approaches to this problem. First, it is now widely recognized that using experimental data arising from chromatin immunoprecipitation (ChIP) studies can enrich for regulatory regions. The new oPOSSUM system features the capacity to process user-supplied DNA sequences. Although the cases presented in this report focus on TF-specific ChIP-Seq data, users may analyze any set of sequences they generate. Research that combines subsets of gene expression, epigenetic marks, and TF binding data could yield interesting sequence sets for analysis with oPOSSUM-3. For gene-based analysis, principally used by researchers working with gene expression data, the use of phylogenetic footprinting to restrict analysis remains the most common approach. The transition from pairwise alignment analysis to multiple sequence alignment analysis presents key benefits and raises challenges. Multiple sequence alignment approaches to phylogenetic footprinting can be performed for a dramatically higher portion of genes (a 66% increase observed in this study) and lead to improved alignment quality (Kumar and Filipski 2007). There remains a need to refine the multiple species footprinting approach using more advanced methods that incorporate motif conservation as well as sequence conservation. The recently published MotEvo method may address this challenge (Arnold *et al.* 2012). In the near future, a broad and systematic benchmarking study of multiple species phylogenetic footprinting procedures for motif enrichment and *de novo* motif discovery would advance the field.

Although running analyses on oPOSSUM-3 is relatively straightforward, interpretation of results requires some consideration, as expected for any over-representation analysis. First, as shown in the analysis of the effects of background GC-content, one should be aware of nucleotide composition differences between the foreground target set and the background set, as such differences can bias the TFBS profile enrichment scores. oPOSSUM-3 reports the GC-content of the foreground and background sets used in each analysis to empower users to make such assessments. A second consideration is the selection of the enrichment scoring method. While in some cases the profile of a mediating TF scores high on both Z-score and Fisher score rankings, empirical observations indicate that the two metrics can differ substantially. The differences reflect properties of the frequency of predicted TFBS. Fisher scores indicate the number of sequences or genes containing a predicted TFBS, whereas Z-scores reflect the frequency of the predicted TFBSs. The KS scores, which are new to oPOSSUM, measure the tendency for the predicted TF binding sites to cluster near a position of maximum confidence in the peak, or near the peak center if the user does not provide a position of confidence.

There are opportunities to enhance oPOSSUM-3 in the future. The oPOSSUM system should be linked to a curated database of TFs, such as DBD or TFCat (Fulton *et al.* 2009; Wilson *et al.* 2008). By linking the systems, we can indicate which genes in the foreground target set are encoding TFs and therefore which genes may be candidates for participation in regulatory feedback loops. Based on similar considerations, the regulatory ncRNA genes should be reported. Finally, the system can be modified to incorporate automated methods to correct for biases in the gene or sequence foreground sets that are not represented in the default background set.

Ultimately, we expect the oPOSSUM-3 system to be a convenient and useful tool for transcriptional regulation analysis, providing researchers with insight into the transcription factors acting on their sets of genes or sequences. The user-friendly interface provides researchers with access to a powerful bioinformatics tool.

## Supplementary Material

Supporting Information
