# Supplementary material for: oPOSSUM-3: Advanced Analysis of Regulatory Motif Over-Representation Across Genes or ChIP-Seq Datasets
Source: G3 (Bethesda). 2012 Sep 1;2(9):987–1002. doi: 10.1534/g3.112.003202 (PMC3429929; doi:10.1534/g3.112.003202)
Supplement: Supporting Information [file supp_2_9_987__index.html]

Supporting Information 

# oPOSSUM-3: Advanced Analysis of Regulatory Motif Over-Representation Across Genes or ChIP-Seq Datasets

## Supporting Information for Kwon *et al.*, 2012

**Files in this Data Supplement:**

- Supporting Information - Figures S1-S8 and Table S1 (PDF, 2.8 MB)
- Table S1 - Muscle reference gene collection (PDF, 59 KB)
- Figure S1 - oPOSSUM-specific JASPAR PENDING collection (PDF, 674 KB)
- Figure S2 - Predicted Nfe2L2 binding site distributions between ChIP-Seq foreground and background sequences (PDF, 193 KB)
- Figure S3 - Defining conserved regions (PDF, 316 KB)
- Figure S4 - TFBS clustering algorithm pseudocode (PDF, 691 KB)
- Figure S5 - oPOSSUM system provisions for species with operon structures (PDF, 191 KB)
- Figure S6 - The range of enrichment scores increases as the number of foreground sequences increases (PDF, 294 KB)
- Figure S7 - Fisher scores vs. Z-scores from oPOSSUM analysis on sequence-based data (PDF, 395 KB)
- Figure S8 - Applied thresholds for KS scores from sequence-based data (PDF, 409 KB)
