# Supplementary material for: oPOSSUM-3: Advanced Analysis of Regulatory Motif Over-Representation Across Genes or ChIP-Seq Datasets
Source: G3 (Bethesda). 2012 Sep 1;2(9):987–1002. doi: 10.1534/g3.112.003202 (PMC3429929; doi:10.1534/g3.112.003202)
Supplement: Supporting Information [file supp_2.9.987_003202SI.pdf]

A. daf-19

```
A [ 1  0  3  6  0  0 21  0  6  1  1 20 22  0]
C [ 0  1  4  3 21 17  0  0  0  1 15  0  0 21]
G [21  0  0  3  0  0  0  0 16 20  0  1  0  0]
T [ 0 21 15 10  1  5  1 22  0  0  6  1  0  1]
```

B. Rfx1_1

```
A [5 10  6  3 11 13  3  4  4 11  0  3 30 32  0 11 11]
C [5  4  4  2 10  1 25 14  2  3  0 16  0  0 32  9  3]
G [6  8 22  7  2 17  1  7  0 17 32  3  1  0  0  7  5]
T [8  6  0 20  9  1  3  7  6  1  0 10  1  0  0  4 10]
```

C. Rfx1_2

```
A [5 10  6  3 11 13  3  4  9  4 11  0  3 30 32  0 11 11]
C [5  4  4  2 10  1 25 14  3  2  3  0 16  0  0 32  9  3]
G [6  8 22  7  2 17  1  7  2  0 17 32  3  1  0  0  7  5]
T [8  6  0 20  9  1  3  7  2 10  1  0 10  1  0  0  4 10]
```

D. Nfe2l2

```
A [288   0   0 430  14  58  31 345   3   3 361]
C [ 24   2   0   0 304  13 325   2   3 418  15]
G [112   0 419   0  75  12  31  49 424   6  17]
T [  6 428  11   0  37 347  43  34   0   3  37]
```

Figure S1 oPOSSUM-specific JASPAR PENDING collection. PFMs for daf-19, Rfx1_1, Rfx1_2 and Nfe2L2 are included.

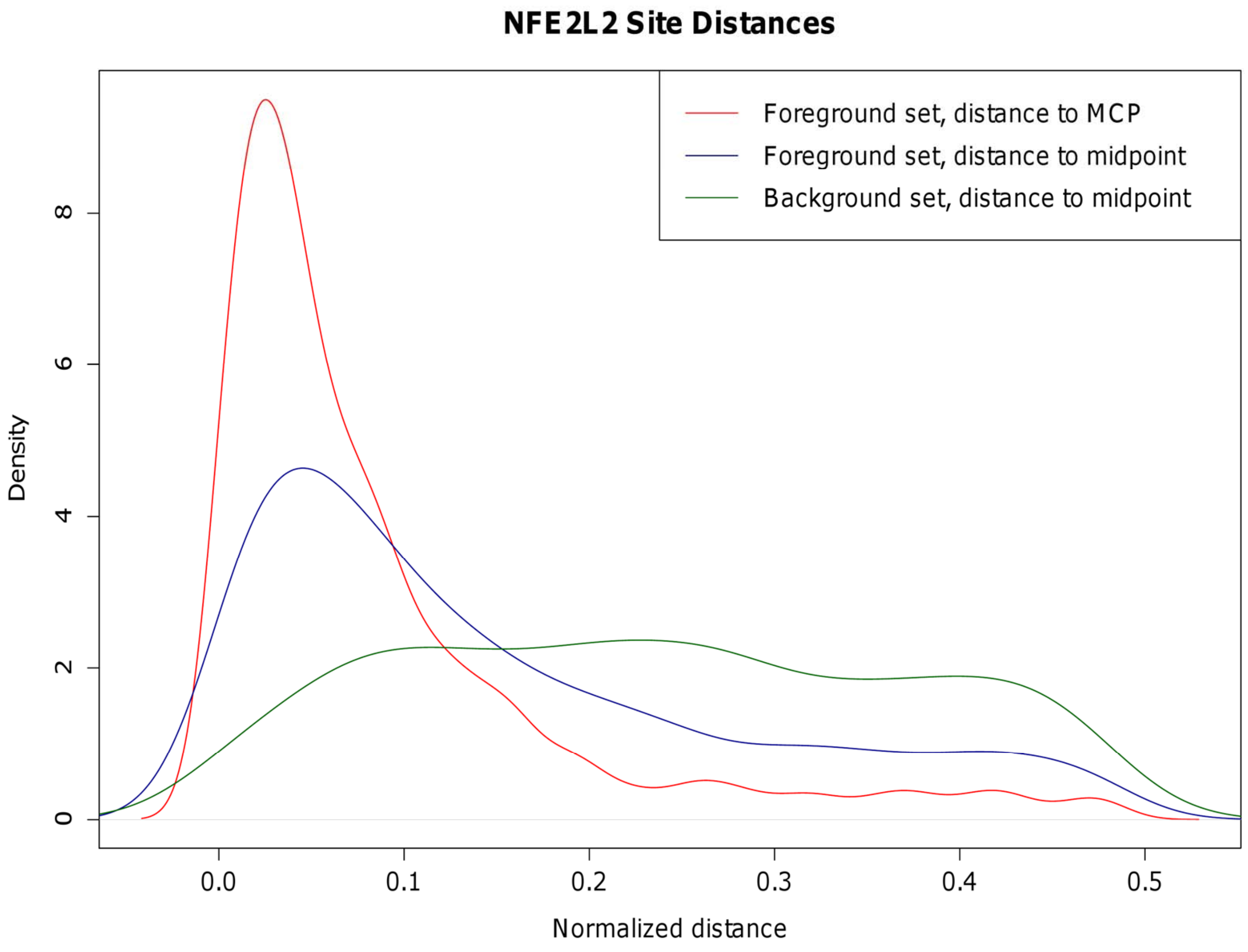

Figure S2 Predicted Nfe2L2 binding site distributions between ChIP-Seq foreground and background sequences. The red distribution is the distances between predicted Nfe2L2 sites and the MCP (maximum confidence positions), the blue and green distributions are the distances between Nfe2L2 predicted sites and the middle of the peak, where blue is for foreground peaks and green is for background sequences. Nfe2L2 predicted sites are clustered around the maximum confidence positions in the foreground sequences.

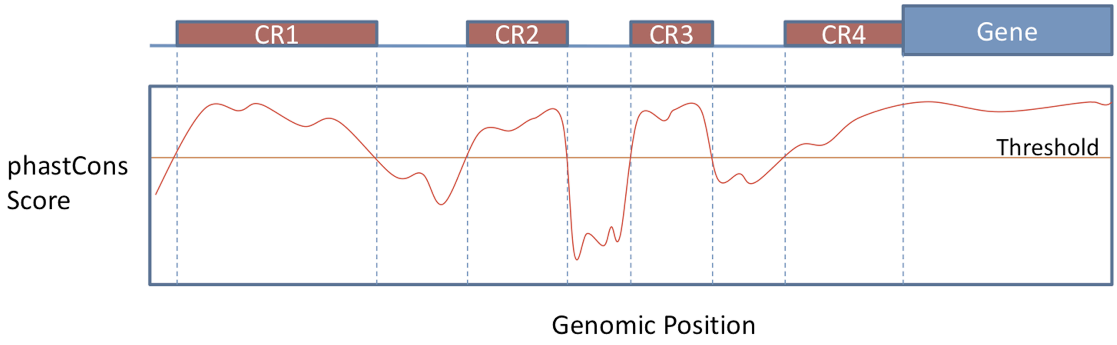

Figure S3 Defining conserved regions. During the oPOSSUM DB build, the phastCons sequence conservation scores from UCSC are retrieved for the pre-defined search region near the transcription start site of each gene. Sub-regions with phastCons scores above the pre-defined threshold are marked as conserved regions, and the TFBS searches are restricted to these sub-regions only.

```
# get all TFBS clusters within each TF family
for each tf_family
  for each tf in tf_family
  search_nearby_for_cluster(tf, new_cluster)
  add new_cluster to cluster_list

# recursively add nearby TFs to form a cluster
function search_nearby_for_cluster(tf, cluster)
{
  # stop if this tf is too far from the cluster being formed
  return if (distance(tf, clustered) > Tmax)

  # go through all nearby neighbours that haven't already been
included
  neighbours = get_neighbours(tf, cluster)
  for each neighbour (neighbours) {
  if (distance(neighbour, cluster) < Tmax)
  add neighbour to cluster

  # check the chain of neighbours and add if they are close
  all_neighbours = search_nearby_for_cluster(neighbour, cluster)
  for each neighbour2 in all_neighbours
  if (distance(neighbour2, cluster) < Tmax)
  add neighbour2 to cluster
}
```

Figure S4 TFBS clustering algorithm pseudocode.

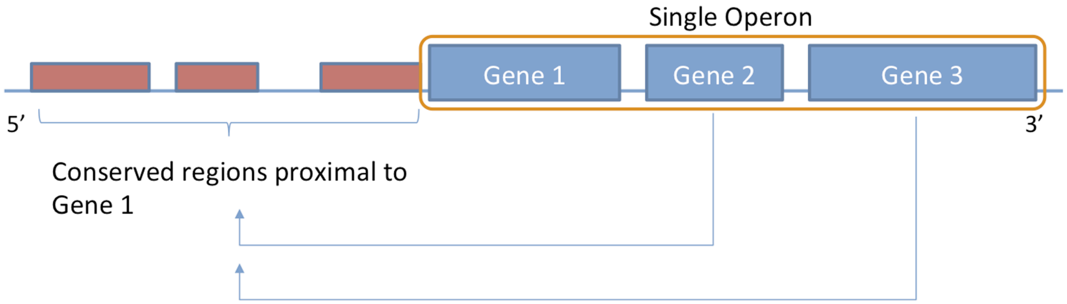

Figure S5 oPOSSUM system provisions for species with operon structures. Nematode operon annotation is retrieved from Wormbase, a central repository for nematodes. If a gene is found to be a member of an operon, the search region is changed to that belonging to the most 5’ gene.

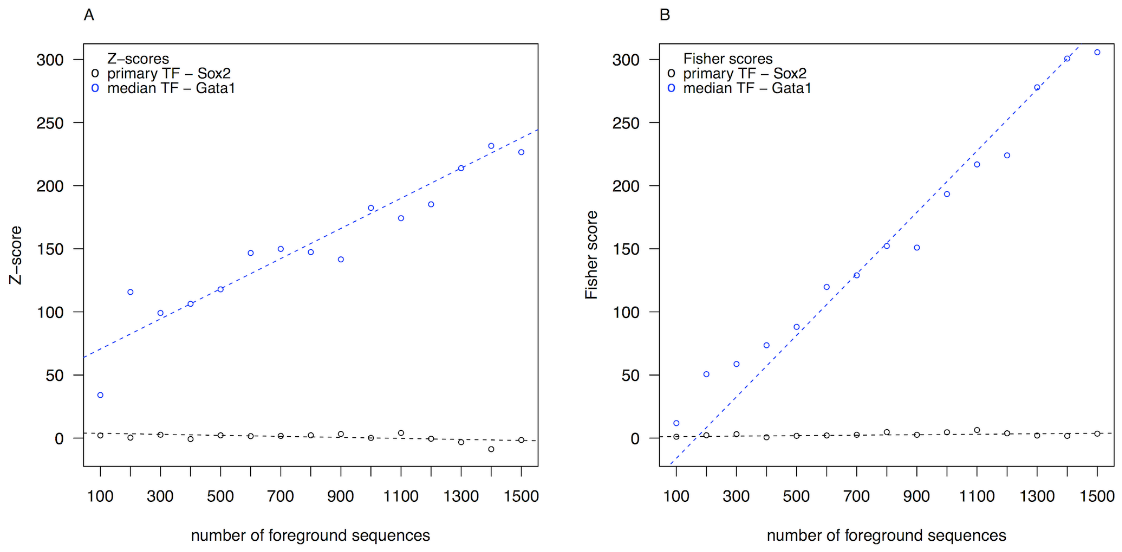

Figure S6 The range of enrichment scores increases as the number of foreground sequences increases. We performed SSA on a series of foregrounds ranging from 100-1500 sequences and corresponding GC composition backgrounds. The impact of foreground size on the two enrichments scores is linear, as shown in (A) Z-score and (B) Fisher score. The plots display scores for i) the primary TF – Sox2, and ii) the median TF – Gata1.

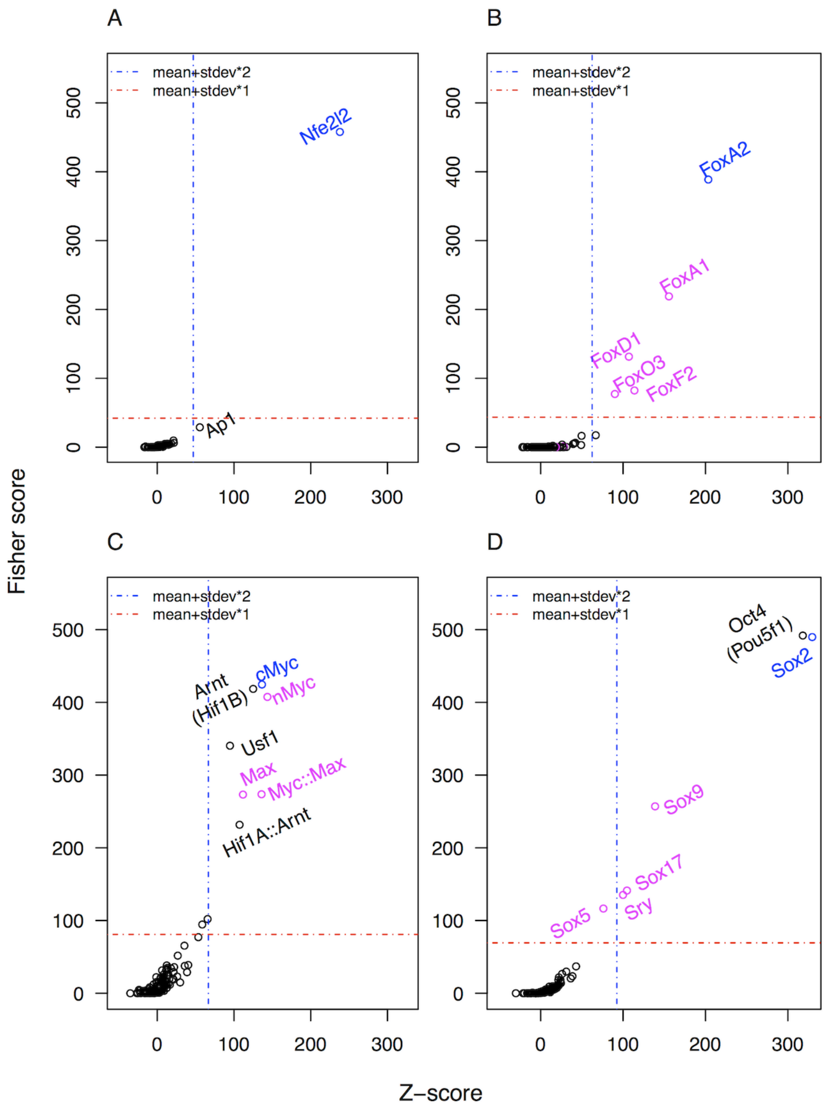

Figure S7 Fisher scores vs. Z-scores from oPOSSUM analysis on sequence-based data. The Fisher and Z- scores in each panel represent the respective enrichment statistic for 116 motifs in selected ChIP-Seq regions per TF of interest, (A) Nfe2L2 (1256 regions), (B) FoxA2 (1200 regions), (C) cMyc (1200 regions), and (D) Sox2 (1200 regions). Blue labels are the target TFs, and pink labels are the TFs related by family to the target TF. The dotted lines are the applied thresholds for the respective enrichment score. TF profiles in the upper-right quadrant are those favoured by both scoring measures. For panel (D), Oct4 and Sox2 share similar binding specificities and are known to interact.

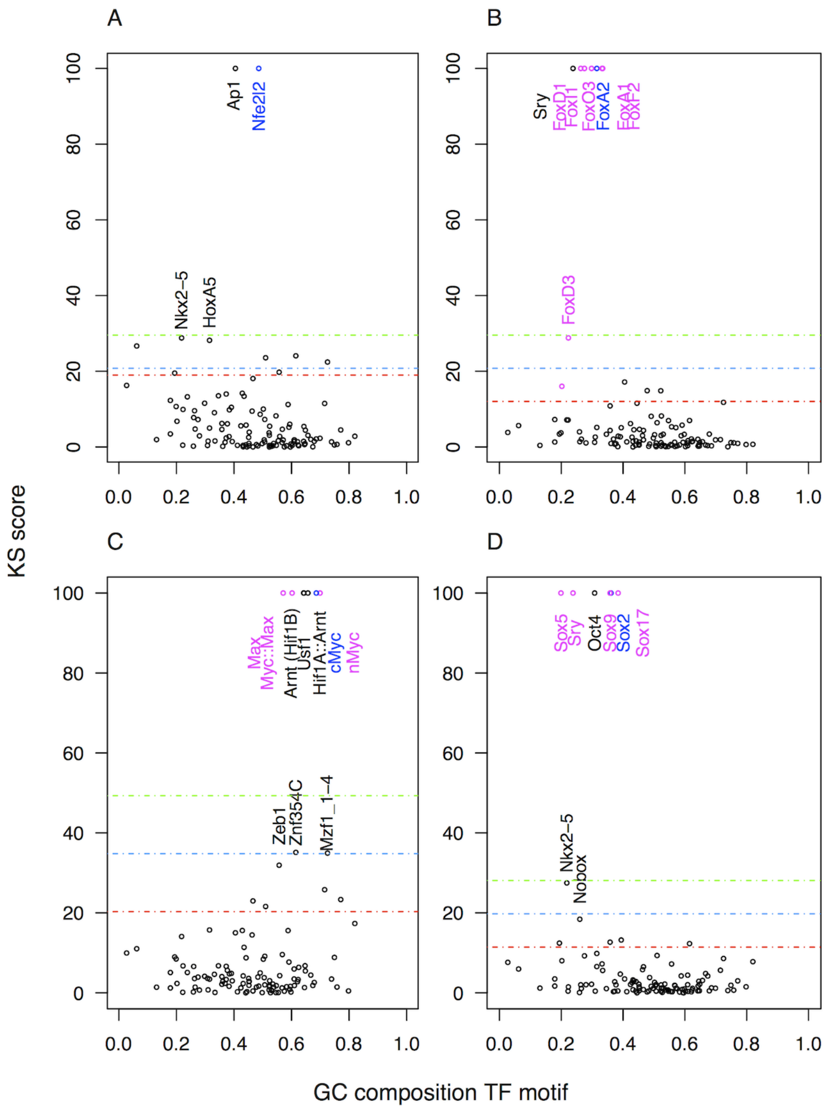

Figure S8 Applied thresholds for KS scores from sequence-based data. The KS scores in each panel represent the respective enrichment statistic for 116 motifs in selected ChIP-Seq regions per TF of interest, (A) Nfe2L2 (1256 regions), (B) FoxA2 (1200 regions), (C) cMyc (1200 regions), and (D) Sox2 (1200 regions). KS scores equal to “Infinite” have been converted to 100 for the sake of visualization. Blue labels are the target TFs, and pink labels are the TFs related by family to the target TF. The dotted lines are potential thresholds (the mean plus 2x - red, 4x - blue and 6x - green standard deviations) for the KS score.

Table S1 Muscle reference gene collection. These are human genes known to be muscle-specific, and have been found to be associated with muscle-specific enhancer regions.

Gene Symbol	Ensembl ID	Reference (PMID)
Aldoa	ENSG00000149925	10369770, 7473711
DMD	ENSG00000198947	11259421
MB	ENSG00000198125	11279187
MEF2C	ENSG00000081189	11714687
MYH4	ENSG00000141048	10329954
MYH3	ENSG00000109063	11971910
SLC2A4	ENSG00000181856	12893821
ACHA	ENSG00000138435	9571041
CHRNB1	ENSG00000170175	9571041
ACHG	ENSG00000196811	9571041
ACHD	ENSG00000135902	9571041
ACHE	ENSG00000108556	9571041
ACTC	ENSG00000159251	9571041
CKM	ENSG00000104879	9571041
DES	ENSG00000175084	9571041
MYF6	ENSG00000111046	9571041
MYOD	ENSG00000129152	9571041
MYOG	ENSG00000122180	9571041
MYL1	ENSG00000168530	9571041
MYL4	ENSG00000198336	9571041
TNCC1	ENSG00000114854	9571041
TNNI1	ENSG00000159173	9571041

MYH7	ENSG00000092054	9571041
MYH6	ENSG00000197616	9571041
ACTC1	ENSG00000143632	9571041
