# Supplementary material for: oPOSSUM-3: Advanced Analysis of Regulatory Motif Over-Representation Across Genes or ChIP-Seq Datasets
Source: G3 (Bethesda). 2012 Sep 1;2(9):987–1002. doi: 10.1534/g3.112.003202 (PMC3429929; doi:10.1534/g3.112.003202)
Supplement: Supporting Information [file supp_2.9.987_TableS1.pdf]

Table S1 Muscle reference gene collection. These are human genes known to be muscle-specific, and have been found to be associated with muscle-specific enhancer regions.

Gene Symbol	Ensembl ID	Reference (PMID)
Aldoa	ENSG00000149925	10369770, 7473711
DMD	ENSG00000198947	11259421
MB	ENSG00000198125	11279187
MEF2C	ENSG00000081189	11714687
MYH4	ENSG00000141048	10329954
MYH3	ENSG00000109063	11971910
SLC2A4	ENSG00000181856	12893821
ACHA	ENSG00000138435	9571041
CHRNB1	ENSG00000170175	9571041
ACHG	ENSG00000196811	9571041
ACHD	ENSG00000135902	9571041
ACHE	ENSG00000108556	9571041
ACTC	ENSG00000159251	9571041
CKM	ENSG00000104879	9571041
DES	ENSG00000175084	9571041
MYF6	ENSG00000111046	9571041
MYOD	ENSG00000129152	9571041
MYOG	ENSG00000122180	9571041
MYL1	ENSG00000168530	9571041
MYL4	ENSG00000198336	9571041
TNCC1	ENSG00000114854	9571041
TNNI1	ENSG00000159173	9571041

MYH7	ENSG00000092054	9571041
MYH6	ENSG00000197616	9571041
ACTC1	ENSG00000143632	9571041
