# Supplementary material for: oPOSSUM-3: Advanced Analysis of Regulatory Motif Over-Representation Across Genes or ChIP-Seq Datasets
Source: G3 (Bethesda). 2012 Sep 1;2(9):987–1002. doi: 10.1534/g3.112.003202 (PMC3429929; doi:10.1534/g3.112.003202)
Supplement: Supporting Information [file supp_2.9.987_FigureS1.pdf]

A. daf-19

```
A [ 1  0  3  6  0  0 21  0  6  1  1 20 22  0]
C [ 0  1  4  3 21 17  0  0  0  1 15  0  0 21]
G [21  0  0  3  0  0  0  0 16 20  0  1  0  0]
T [ 0 21 15 10  1  5  1 22  0  0  6  1  0  1]
```

B. Rfx1_1

```
A [5 10  6  3 11 13  3  4  4 11  0  3 30 32  0 11 11]
C [5  4  4  2 10  1 25 14  2  3  0 16  0  0 32  9  3]
G [6  8 22  7  2 17  1  7  0 17 32  3  1  0  0  7  5]
T [8  6  0 20  9  1  3  7  6  1  0 10  1  0  0  4 10]
```

C. Rfx1_2

```
A [5 10  6  3 11 13  3  4  9  4 11  0  3 30 32  0 11 11]
C [5  4  4  2 10  1 25 14  3  2  3  0 16  0  0 32  9  3]
G [6  8 22  7  2 17  1  7  2  0 17 32  3  1  0  0  7  5]
T [8  6  0 20  9  1  3  7  2 10  1  0 10  1  0  0  4 10]
```

D. Nfe2l2

```
A [288   0   0 430  14  58  31 345   3   3 361]
C [ 24   2   0   0 304  13 325   2   3 418  15]
G [112   0 419   0  75  12  31  49 424   6  17]
T [  6 428  11   0  37 347  43  34   0   3  37]
```

Figure S1 oPOSSUM-specific JASPAR PENDING collection. PFMs for daf-19, Rfx1_1, Rfx1_2 and Nfe2L2 are included.
