# Supplementary material for: oPOSSUM-3: Advanced Analysis of Regulatory Motif Over-Representation Across Genes or ChIP-Seq Datasets
Source: G3 (Bethesda). 2012 Sep 1;2(9):987–1002. doi: 10.1534/g3.112.003202 (PMC3429929; doi:10.1534/g3.112.003202)
Supplement: Supporting Information [file supp_2.9.987_FigureS2.pdf]

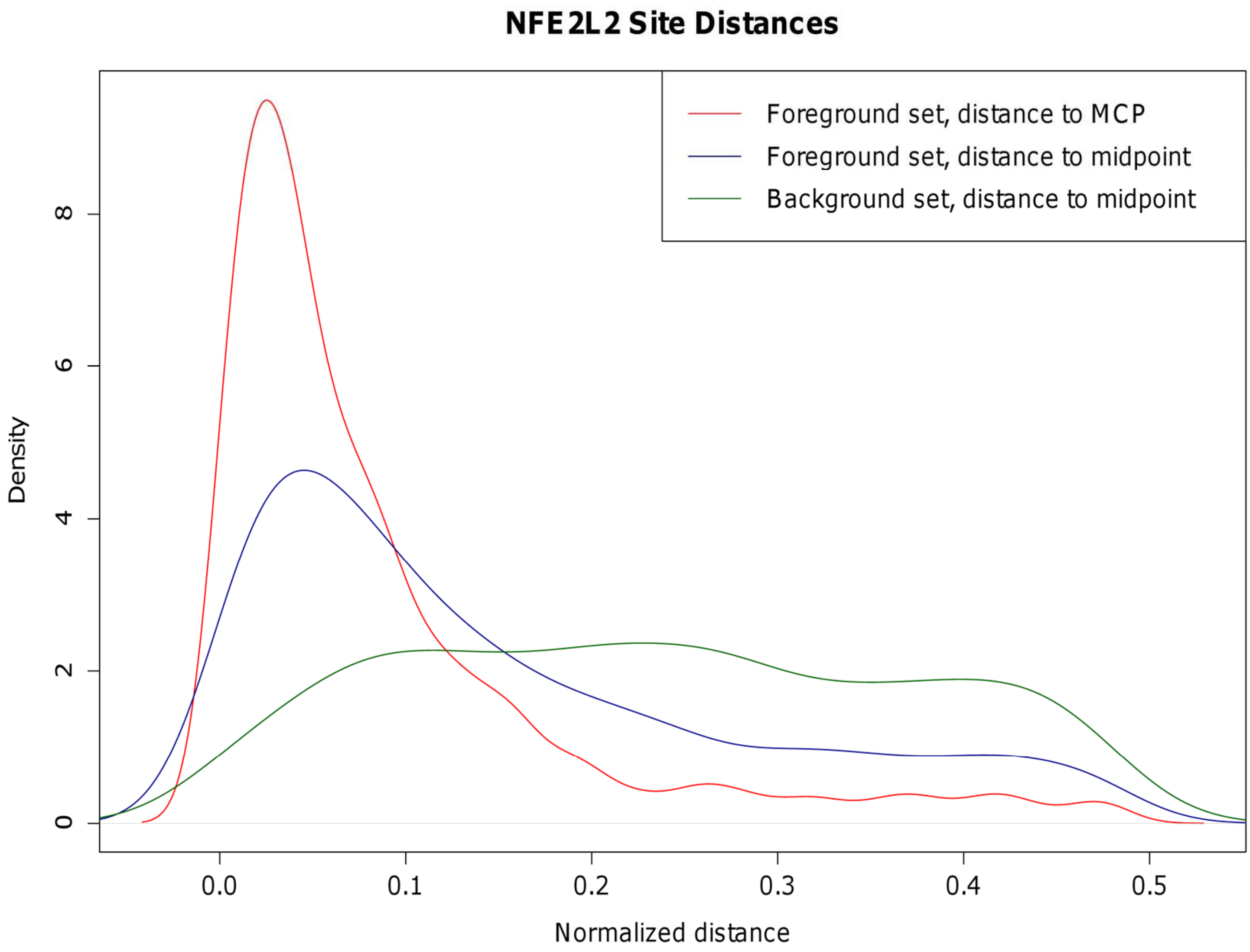

Figure S2 Predicted Nfe2L2 binding site distributions between ChIP-Seq foreground and background sequences. The red distribution is the distances between predicted Nfe2L2 sites and the MCP (maximum confidence positions), the blue and green distributions are the distances between Nfe2L2 predicted sites and the middle of the peak, where blue is for foreground peaks and green is for background sequences. Nfe2L2 predicted sites are clustered around the maximum confidence positions in the foreground sequences.
