# Supplementary material for: oPOSSUM-3: Advanced Analysis of Regulatory Motif Over-Representation Across Genes or ChIP-Seq Datasets
Source: G3 (Bethesda). 2012 Sep 1;2(9):987–1002. doi: 10.1534/g3.112.003202 (PMC3429929; doi:10.1534/g3.112.003202)
Supplement: Supporting Information [file supp_2.9.987_FigureS3.pdf]

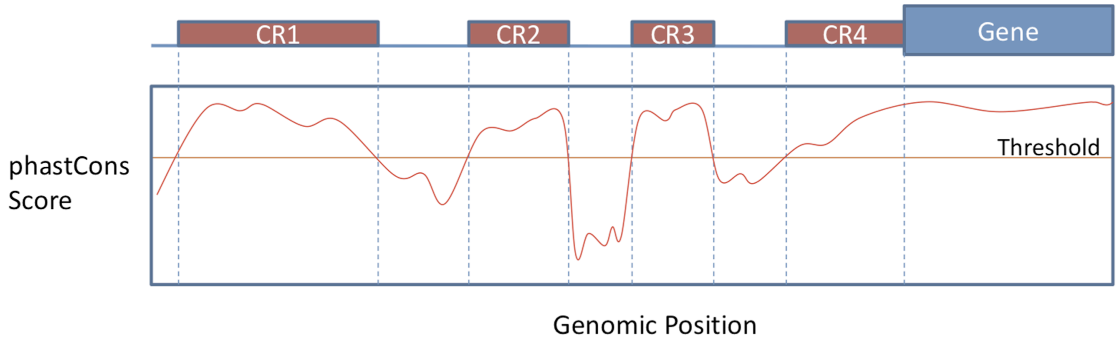

Figure S3 Defining conserved regions. During the oPOSSUM DB build, the phastCons sequence conservation scores from UCSC are retrieved for the pre-defined search region near the transcription start site of each gene. Sub-regions with phastCons scores above the pre-defined threshold are marked as conserved regions, and the TFBS searches are restricted to these sub-regions only.
