# Supplementary material for: oPOSSUM-3: Advanced Analysis of Regulatory Motif Over-Representation Across Genes or ChIP-Seq Datasets
Source: G3 (Bethesda). 2012 Sep 1;2(9):987–1002. doi: 10.1534/g3.112.003202 (PMC3429929; doi:10.1534/g3.112.003202)
Supplement: Supporting Information [file supp_2.9.987_FigureS4.pdf]

```
# get all TFBS clusters within each TF family
for each tf_family
  for each tf in tf_family
  search_nearby_for_cluster(tf, new_cluster)
  add new_cluster to cluster_list

# recursively add nearby TFs to form a cluster
function search_nearby_for_cluster(tf, cluster)
{
  # stop if this tf is too far from the cluster being formed
  return if (distance(tf, clustered) > Tmax)

  # go through all nearby neighbours that haven’t already been
included
  neighbours = get_neighbours(tf, cluster)
  for each neighbour (neighbours) {
  if (distance(neighbour, cluster) < Tmax)
  add neighbour to cluster

  # check the chain of neighbours and add if they are close
  all_neighbours = search_nearby_for_cluster(neighbour, cluster)
  for each neighbour2 in all_neighbours
  if (distance(neighbour2, cluster) < Tmax)
  add neighbour2 to cluster
}
```

Figure S4 TFBS clustering algorithm pseudocode.
