# Supplementary material for: oPOSSUM-3: Advanced Analysis of Regulatory Motif Over-Representation Across Genes or ChIP-Seq Datasets
Source: G3 (Bethesda). 2012 Sep 1;2(9):987–1002. doi: 10.1534/g3.112.003202 (PMC3429929; doi:10.1534/g3.112.003202)
Supplement: Supporting Information [file supp_2.9.987_FigureS5.pdf]

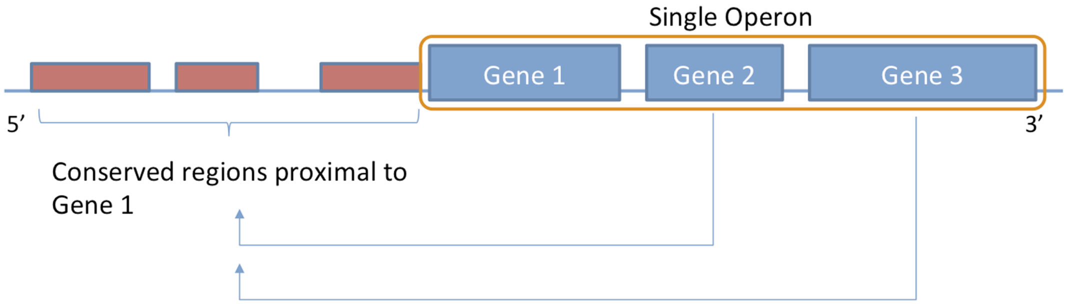

Figure S5 oPOSSUM system provisions for species with operon structures. Nematode operon annotation is retrieved from Wormbase, a central repository for nematodes. If a gene is found to be a member of an operon, the search region is changed to that belonging to the most 5’ gene.
