# Supplementary material for: oPOSSUM-3: Advanced Analysis of Regulatory Motif Over-Representation Across Genes or ChIP-Seq Datasets
Source: G3 (Bethesda). 2012 Sep 1;2(9):987–1002. doi: 10.1534/g3.112.003202 (PMC3429929; doi:10.1534/g3.112.003202)
Supplement: Supporting Information [file supp_2.9.987_FigureS6.pdf]

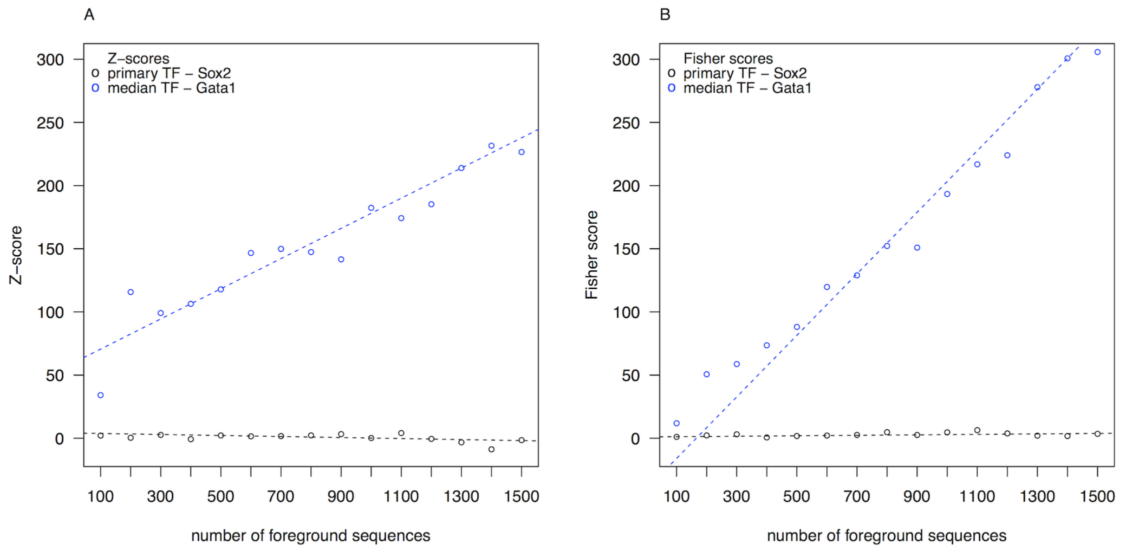

Figure S6 The range of enrichment scores increases as the number of foreground sequences increases. We performed SSA on a series of foregrounds ranging from 100-1500 sequences and corresponding GC composition backgrounds. The impact of foreground size on the two enrichments scores is linear, as shown in (A) Z-score and (B) Fisher score. The plots display scores for i) the primary TF – Sox2, and ii) the median TF – Gata1.
