# Supplementary material for: oPOSSUM-3: Advanced Analysis of Regulatory Motif Over-Representation Across Genes or ChIP-Seq Datasets
Source: G3 (Bethesda). 2012 Sep 1;2(9):987–1002. doi: 10.1534/g3.112.003202 (PMC3429929; doi:10.1534/g3.112.003202)
Supplement: Supporting Information [file supp_2.9.987_FigureS7.pdf]

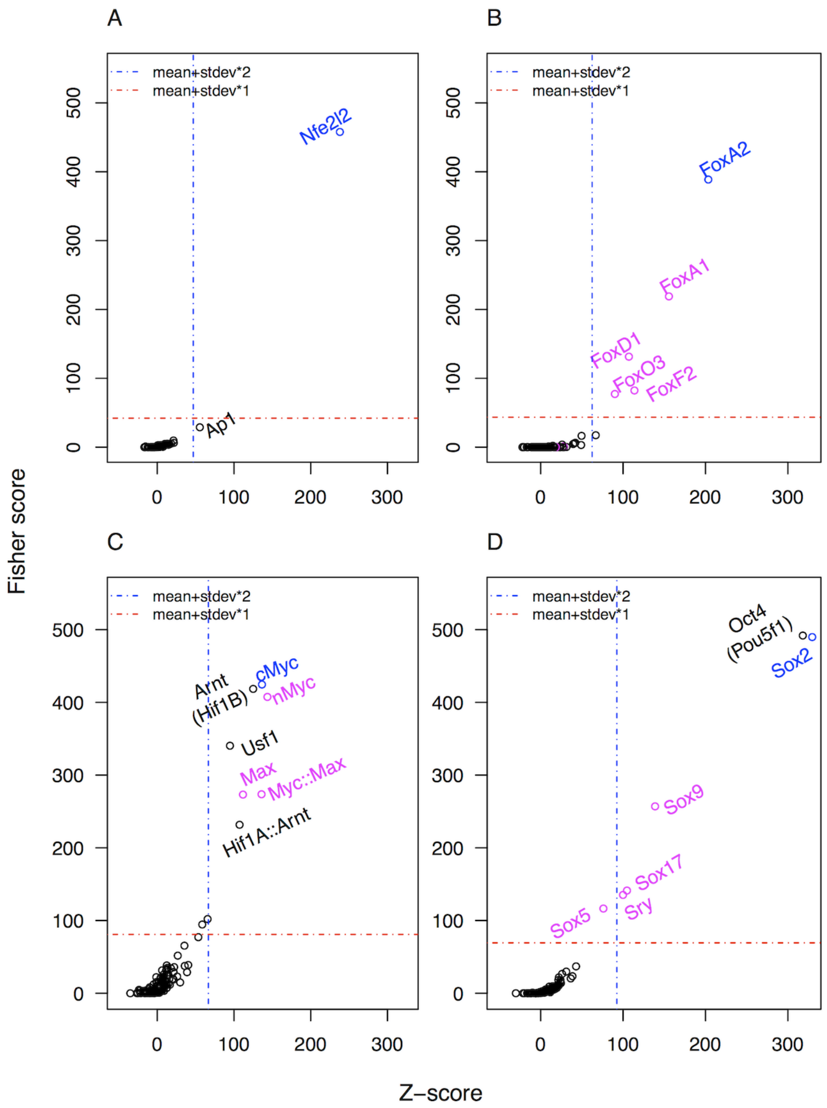

Figure S7 Fisher scores vs. Z-scores from oPOSSUM analysis on sequence-based data. The Fisher and Z- scores in each panel represent the respective enrichment statistic for 116 motifs in selected ChIP-Seq regions per TF of interest, (A) Nfe2L2 (1256 regions), (B) FoxA2 (1200 regions), (C) cMyc (1200 regions), and (D) Sox2 (1200 regions). Blue labels are the target TFs, and pink labels are the TFs related by family to the target TF. The dotted lines are the applied thresholds for the respective enrichment score. TF profiles in the upper-right quadrant are those favoured by both scoring measures. For panel (D), Oct4 and Sox2 share similar binding specificities and are known to interact.
