# Supplementary material for: oPOSSUM-3: Advanced Analysis of Regulatory Motif Over-Representation Across Genes or ChIP-Seq Datasets
Source: G3 (Bethesda). 2012 Sep 1;2(9):987–1002. doi: 10.1534/g3.112.003202 (PMC3429929; doi:10.1534/g3.112.003202)
Supplement: Supporting Information [file supp_2.9.987_FigureS8.pdf]

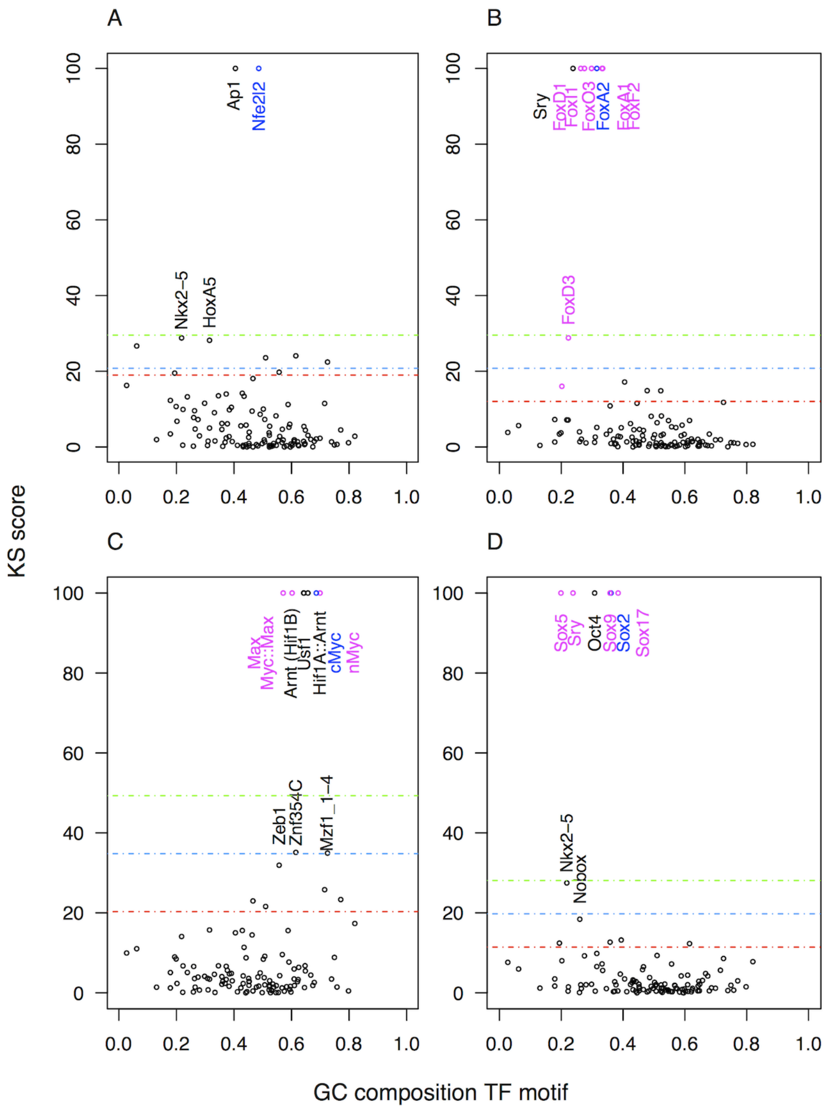

Figure S8 Applied thresholds for KS scores from sequence-based data. The KS scores in each panel represent the respective enrichment statistic for 116 motifs in selected ChIP-Seq regions per TF of interest, (A) Nfe2L2 (1256 regions), (B) FoxA2 (1200 regions), (C) cMyc (1200 regions), and (D) Sox2 (1200 regions). KS scores equal to “Infinite” have been converted to 100 for the sake of visualization. Blue labels are the target TFs, and pink labels are the TFs related by family to the target TF. The dotted lines are potential thresholds (the mean plus 2x - red, 4x - blue and 6x - green standard deviations) for the KS score.
